# Roles of RNA Modifications in Diverse Cellular Functions

**DOI:** 10.3389/fcell.2022.828683

**Published:** 2022-03-08

**Authors:** Emma Wilkinson, Yan-Hong Cui, Yu-Ying He

**Affiliations:** ^1^ Department of Medicine, Section of Dermatology, University of Chicago, Chicago, IL, United States; ^2^ Committee on Cancer Biology, University of Chicago, Chicago, IL, United States

**Keywords:** m^6^A, m^5^C, m^1^A, epitranscriptomics, cellular functions, therapeutics

## Abstract

Chemical modifications of RNA molecules regulate both RNA metabolism and fate. The deposition and function of these modifications are mediated by the actions of writer, reader, and eraser proteins. At the cellular level, RNA modifications regulate several cellular processes including cell death, proliferation, senescence, differentiation, migration, metabolism, autophagy, the DNA damage response, and liquid-liquid phase separation. Emerging evidence demonstrates that RNA modifications play active roles in the physiology and etiology of multiple diseases due to their pervasive roles in cellular functions. Here, we will summarize recent advances in the regulatory and functional role of RNA modifications in these cellular functions, emphasizing the context-specific roles of RNA modifications in mammalian systems. As m^6^A is the best studied RNA modification in biological processes, this review will summarize the emerging advances on the diverse roles of m^6^A in cellular functions. In addition, we will also provide an overview for the cellular functions of other RNA modifications, including m^5^C and m^1^A. Furthermore, we will also discuss the roles of RNA modifications within the context of disease etiologies and highlight recent advances in the development of therapeutics that target RNA modifications. Elucidating these context-specific functions will increase our understanding of how these modifications become dysregulated during disease pathogenesis and may provide new opportunities for improving disease prevention and therapy by targeting these pathways.

## Introduction

Many RNA modifications are reversible modifications that are deposited onto RNA molecules, including mRNAs, tRNAs, rRNAs, and non-coding RNAs. To date, over 100 RNA modifications have been identified, including m^6^A, m^5^C, and m^1^A on mRNA (Roundtree et al., 2017). Of these modifications, N^6^-methyladenosine (m^6^A) is the most abundant internal mRNA modification (Roundtree et al., 2017). Since m^6^A is the best-studied mRNA modification, we will focus on the cellular functions of m^6^A in mammalian systems in this review. Other RNA modifications, such as m^5^C and m^1^A, have been studied in the context of cellular processes as well, and will be summarized here. The role of m^6^A and other RNA modifications in non-mammalian systems, including plants and yeast, is beyond the scope of this review and is detailed elsewhere (Schwartz et al., 2013; Shen et al., 2019; Yue et al., 2019).

**FIGURE 1:**
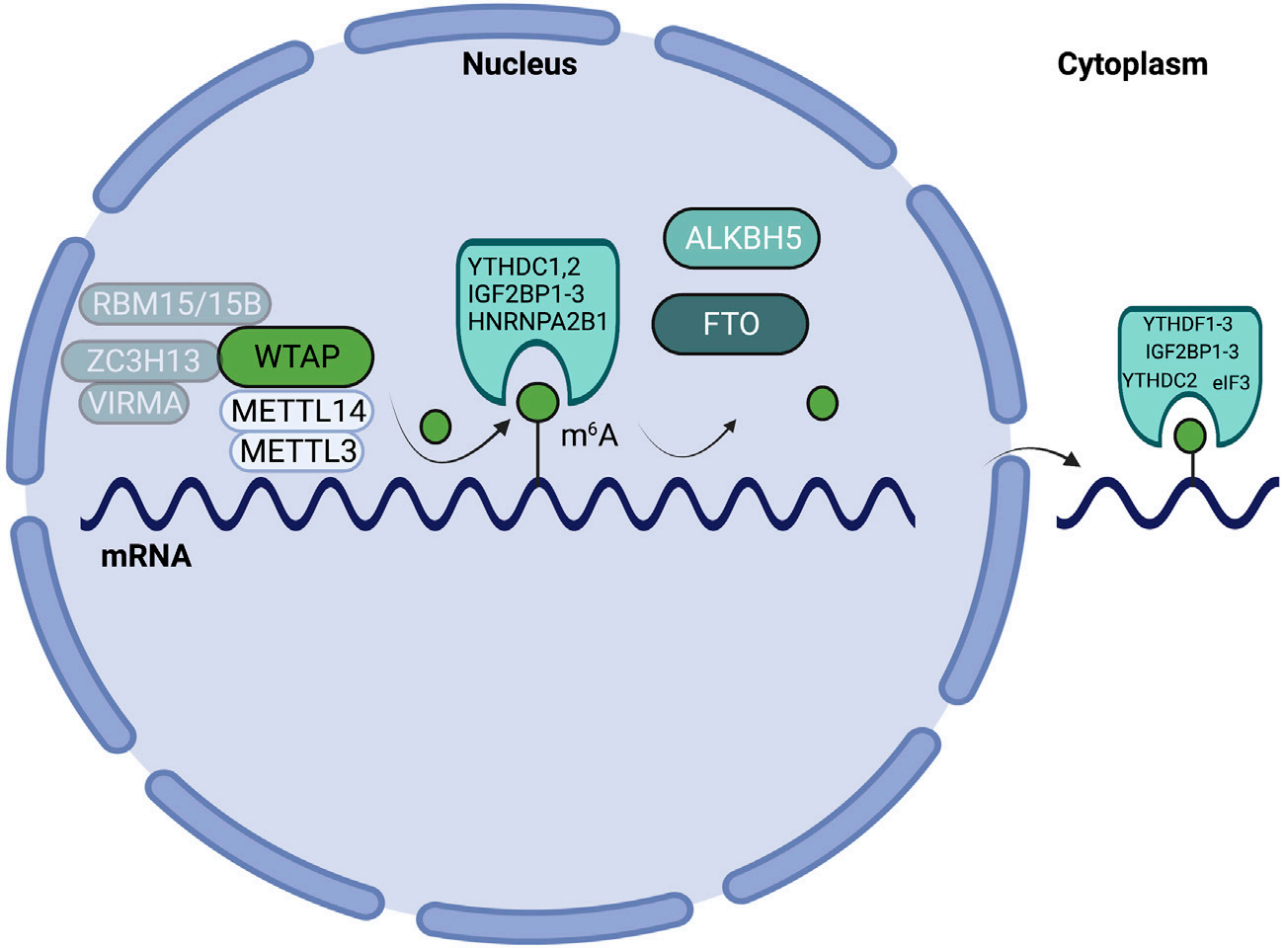
Schematic highlighting m^6^A writers, erasers, and readers on mRNA. m^6^A patterning on mRNA is mediated by the actions of writers (METTL3, METTL14, WTAP, VIRMA/KIAA1429, RBM15/15B, ZC3H13), erasers (ALKBH5, FTO), and reader enzymes (YTHDF1-3, YTHDC1/2, IGF2BP1-3, HNRNPA2B1, and eIF3).

RNA modifications are deposited onto, and erased from, RNA molecules through the actions of writer and eraser enzymes. m^6^A writers and cofactors include METTL3, METTL14, WTAP, VIRMA/KIAA1429, RBM15/15B, ZC3H13, and METTL16 (Yang et al., 2018). Together, METTL3, METTL14, WTAP, VIRMA/KIAA1429, RBM15/15B, and ZC3H13 make up the methyltransferase complex (MTC) (Yang et al., 2018). Within the MTC, METTL3 serves as the catalytic subunit, while METTL14 serves as the RNA-binding subunit (Yang et al., 2018). m^6^A erasers include FTO and ALKBH5 (Yang et al., 2018). RNA modifications influence various mechanisms of RNA metabolism, including nuclear processing, mRNA decay, and translation, through the action of reader enzymes (Roundtree et al., 2017). m^6^A readers include YTHDF1-3, YTHDC1-2, IGF2BP1-3, HNRNPA2B1, and eIF3 (Meyer et al., 2015; Yang et al., 2018). m^6^A mRNA writer, eraser and reader proteins are highlighted in Figure 1. While the writers and erasers can install or remove modifications in RNAs, it is the regulatory effect of the readers that ultimately controls the RNA fate and gene expression.

Another modification on mRNA, tRNA, and other non-coding RNAs, is 5-methylcytosine (m^5^C). m^5^C writers include DNMT2 and the NSUN (NSUN1-7) family proteins (Xue et al., 2020). The NSUN proteins contain an RNA recognition motif and a catalytic core that houses S-adenosylmethionine (SAM) (Bohnsack et al., 2019). Similarly, DNMT2 contains a catalytic site and a SAM binding site (Xue et al., 2020). The detailed biochemical mechanisms of how m^5^C methyltransferases mediate methyl group transfers are discussed elsewhere (Bohnsack et al., 2019). m^5^C readers, or m^5^C binding-proteins, include ALYFREF and YBX1 (Xue et al., 2020). The TET family of enzymes have been hypothesized to serve as m^5^C erasers (Xue et al., 2020). The function of m^5^C in regulating RNA metabolism and expression is summarized elsewhere (Xue et al., 2020).

In addition to m^6^A and m^5^C, *N* ^1^-methyladenosine (m^1^A) is another modification found on mRNA, tRNA, rRNA, and non-coding RNA (Xiong et al., 2018). m^1^A writers include TRMT10C, TRMT6, TRMT61A, and TRMT61B (Xiong et al., 2018). TRMT6/TRMT61A form a heterotetrameric complex wherein TRMT61A functions as the catalytic subunit and TRMT6 is required for its methyltransferase function (Shi et al., 2020; Graille, 2022). m^1^A erasers include ALKBH1 and ALKBH3 (Xiong et al., 2018).

Other RNA modification writer, eraser and reader enzymes are discussed elsewhere (Esteve-Puig et al., 2020). Elucidating the role of RNA modifications in mediating the RNA metabolism of diverse RNA species remains an active area of research.

RNA modifications, and their respective writer, eraser, and reader proteins, also play a role in a number of cellular functions. Here, we summarize the role of RNA modifications in such cellular processes as cell death, proliferation, differentiation, migration, metabolism, autophagy, and liquid-liquid phase separation in mammalian systems. Additionally, we also discuss the cell-type specific targets of these enzymes within these cellular processes.

Due to the pervasive roles of RNA modifications in numerous cellular functions, dysregulated RNA modifications have contributed to the pathogenesis of many diseases and can serve as attractive therapeutic targets due to the reversible nature of these modifications. The role of RNA modifications in diseases is covered in detail elsewhere (Wilkinson et al., 2021). Increasing our knowledge of RNA modifications in cellular processes will increase our understanding of the roles that RNA modifications play in disease etiology and will aid in identifying new therapeutic targets. Clinical success of therapeutics targeting RNA modifications has not been reached and may reflect an incomplete understanding of the role that these modifications play in cellular functions.

## The Role of m^6^A in Diverse Cellular Functions

The writer, eraser, and reader proteins that regulate m^6^A have been well studied in several cellular processes. Of the writer proteins, we will focus on the role of METTL3 and METTL14 in cellular functions, as they are best studied in cellular functions. The role of WTAP and VIRMA is summarized as well.

### Cell Death

Apoptosis is a mechanism of programmed cell death (Elmore, 2007). This process involves coordination and communication across intracellular signaling pathways that ultimately result in the cellular decision to undergo cell death (Elmore, 2007). Apoptosis is initiated in response to pathogens or cellular stressors, immune stimulation, and within embryonic development (Elmore, 2007; Yan et al., 2020). While apoptosis is not the sole mechanism of cell death, it is the best-studied mechanism in the context of m^6^A. The role of m^6^A in specialized forms of cell death remains an active area of research.

#### Writers

The m^6^A writer METTL3 can inhibit apoptosis, as several studies have shown that decreased *Mettl3* expression and methyltransferase activity resulted in increased apoptosis. As previously mentioned, coordinated apoptosis is required for embryonic development (Elmore, 2007; Yan et al., 2020). Accordingly, knockdown of *Mettl3* resulted in decreased m^6^A levels, which increased the mRNA half-lives of neuronal apoptosis-associated genes, including *Dapk1*, *Fadd*, and *Ngfr*, in mouse cerebral granular cells (CGCs) (Wang C.-X. et al., 2018). Increased mRNA half-lives of these genes led to increased apoptosis in CGCs and contributed to severe developmental defects in mouse cerebella (Wang C.-X. et al., 2018).

**FIGURE 2:**
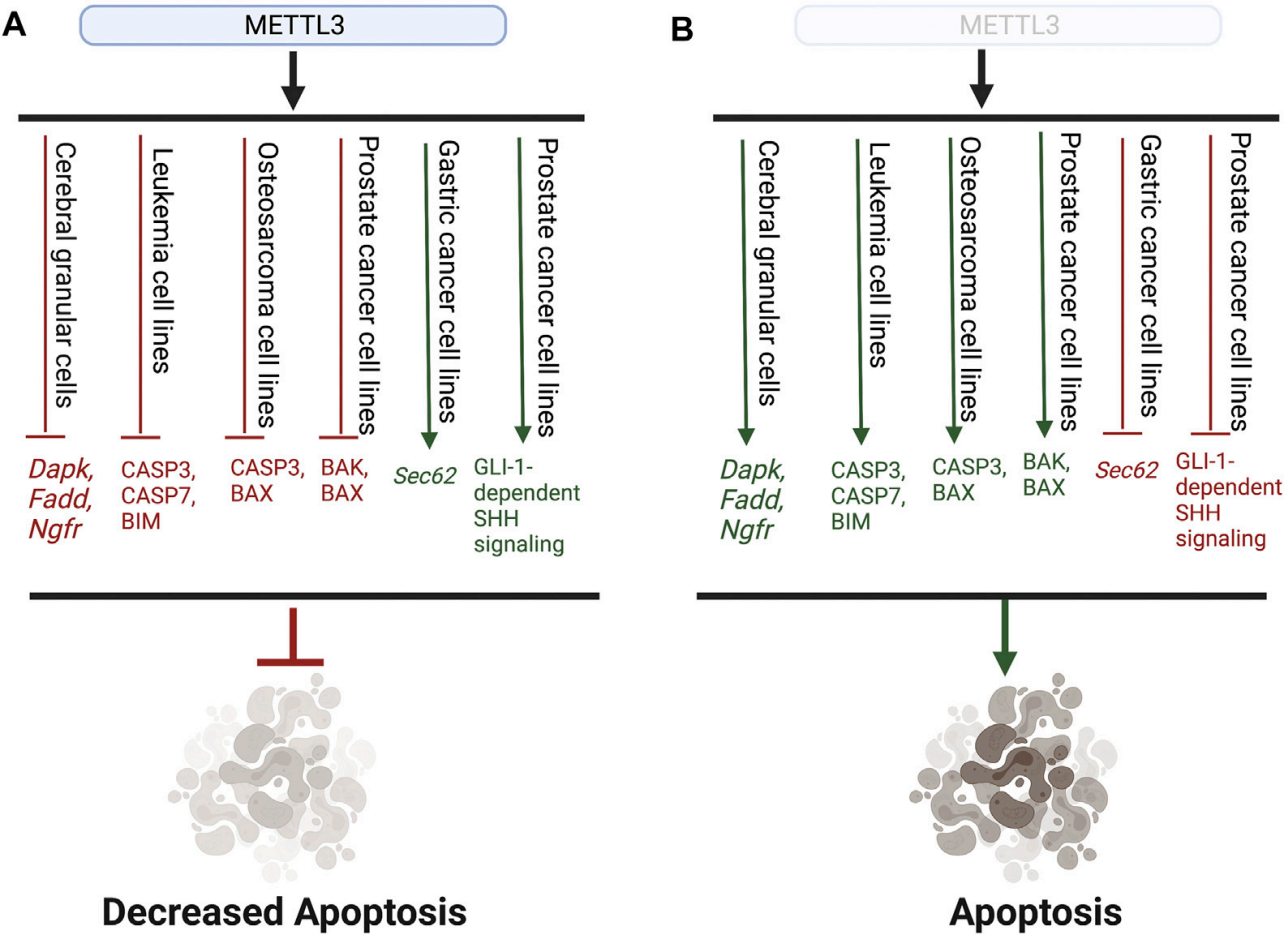
Representative schematic highlighting the role of METTL3 in apoptosis. **(A)** METTL3 prohibits the expression of both pro-apoptotic genes and proteins (red) as well as promotes the expression of anti-apoptotic genes and pathways (green), leading to overall decreased apoptosis. **(B)** Without METTL3 expression, pro-apoptotic genes and proteins are expressed (green), while anti-apoptotic pathways are inhibited, leading to the induction of apoptosis.

Furthermore, several recent studies have established that METTL3 may play an oncogenic role in cell death by negatively regulating and reducing the translation of apoptosis-associated proteins, thereby promoting cell survival (Vu et al., 2017; Choe et al., 2018; Huang et al., 2020). Accordingly, knockdown of *Mettl3* resulted in increased expression of pro-apoptotic proteins in several cancer cell lines, emphasizing that METTL3 can function as a negative regulator of apoptosis (Vu et al., 2017; Zhou et al., 2020). In the MOLM-13 leukemia cell line, knockdown of *Mettl3* increased protein expression of pro-apoptotic proteins CASP3, CASP7, and BIM (Vu et al., 2017). Expression of CASP3 and BAX, other pro-apoptotic proteins, were also increased in *Mettl3* knockdown-osteosarcoma cell lines (Zhou et al., 2020). While it is unclear whether METTL3 regulates apoptosis in an m^6^A-dependent manner, both studies provide evidence that METTL3 may inhibit apoptosis by regulating BCL-2 family proteins and caspase expression (Vu et al., 2017; Zhou et al., 2020). Knockdown of *Mettl3* in prostate cancer cells also increased protein expression of pro-apoptotic proteins BAK and BAX, CASP3 and CASP7 activity, and PARP cleavage (Cai et al., 2019). Additionally, knockdown of *Mettl3* in prostate cancer cells decreased protein expression of anti-apoptotic genes BCL-2 and BCL-XL (Cai et al., 2019). Furthermore, knockdown of *Mettl3* decreased expression of GLI1, a component of the Sonic hedgehog (SHH) signaling pathway, which prostate cancer cells are dependent on for survival (Regl et al., 2002; Chen et al., 2011; Cai et al., 2019). Due to prostate cancer cells’ dependence on SHH signaling, decreased expression of GLI1 deprived prostate cancer cells of SHH signaling and forced apoptosis (Cai et al., 2019). METTL3 regulated the expression of GLI1 in an m^6^A-dependent manner, as expression of GLI1 was rescued by the re-expression of wild-type, but not catalytically-inactive mutant, METTL3 (Cai et al., 2019). In gastric cancer, METTL3 promoted the mRNA stability of *Sec62*, which functions as a negative regulator of apoptosis, in an m^6^A/IGF2BP1 -dependent manner (He et al., 2019). Increased *Sec62* mRNA stability and expression subsequently lead to decreased apoptosis and increased gastric cancer cell survival (He et al., 2019). The role of METTL3 in apoptosis is summarized in Figure 2.

In contrast, the role of METTL14 in mediating apoptosis is not as widely explored and presents an area of research that requires future study. One study found that knockdown of *Mettl14* promoted apoptosis in human AML cell lines (Weng et al., 2018). Mechanistically, METTL14 was found to promote cell survival by promoting the mRNA stability and translation of pro-survival proteins MYB and MYC in an m^6^A-dependent manner (Weng et al., 2018). Therefore, in the absence of METTL14, MYB and MYC expression was decreased, leading to the induction of apoptosis (Weng et al., 2018).

Few studies have explored the role of m^6^A writer-associated protein WTAP in cell death. One study found that WTAP expression was increased upon exposure to hypoxia/reoxygenation (H/R), resulting in the induction of ER stress and apoptosis in cardiomyocyte cells (Wang J. et al., 2021). Accordingly, H/R exposure in *Wtap* knockdown cells abrogated the induction of ER stress and apoptosis, suggesting that WTAP may regulate these processes upon H/R exposure (Wang J. et al., 2021). Mechanistically, WTAP was found to promote the mRNA stability of ER stress-response gene, *Atf4*, in an m^6^A-dependent manner (Wang J. et al., 2021).

VIRMA, another m^6^A writer-associated protein, has not been well-studied in the context of cell death. VIRMA has been found to serve an oncogenic role in several cancers, and was found to promote resistance to apoptosis in HCC (Lan et al., 2019). Mechanistically, VIRMA mediated the deposition of m^6^A onto the 3′-untranslated region (UTR) of *Gata3*, a tumor suppressor, resulting in decreased GATA3 expression and promoted resistance to apoptosis (Lan et al., 2019). The oncogenic role of VIRMA in several cancers is covered elsewhere in detail (Zhu W. et al., 2021).

#### Erasers

The role of the m^6^A eraser ALKBH5 in apoptosis is cell-type dependent. *Alkbh5*-deficient mice exhibited increased apoptosis and developmental defects, potentially through a p53-mediated mechanism that is not entirely understood (Zheng et al., 2013). Conversely, *Alkbh5* knockdown in human ovarian granulosa (hGCs) cells had no effect on the apoptosis rate; rather, knockdown of *Fto* resulted in increased apoptosis in hGCs (Ding et al., 2018).

The role of FTO in apoptosis may also be cell-type-dependent. In leukemia, FTO is preferentially inhibited by R-2-hydroxyglutarate (R-2HG), an oncometabolite produced by mutant IDH1/2 enzymes (Ye et al., 2013; Su et al., 2018). *Fto* depletion decreased the mRNA stability and mRNA expression of downstream targets *Myc* and *Cebpa* through increased m^6^A accumulation (Su et al., 2018). Decreased expression of *Myc* and *Cebpa* resulted in decreased expression of downstream MYC targets, including the E2F transcription factors, which are major regulators of cell cycle, thereby preventing cells from entering the cell cycle and forcing apoptosis or cell cycle arrest (Su et al., 2018). The trend wherein knockdown of *Fto* resulted in increased apoptosis was also seen in breast cancer and melanoma, suggesting that FTO may also serve an oncogenic function in these cancers by inhibiting apoptosis (Yang S. et al., 2019; Niu et al., 2019). In breast cancer, FTO mediated the demethylation of m^6^A at the 3′-UTR of *Bnip3*, a pro-apoptotic gene, resulting in YTHDF2-mediated degradation of the *Bnip3* transcript (Niu et al., 2019). In melanoma, FTO promoted resistance to IFN γ-mediated cell death through m^6^A demethylation of pro-tumorigenic genes *Pdcd-1*, *Cxcr4*, and *Sox10* (Yang S. et al., 2019). Demethylation of *Pdcd-1*, *Cxcr4*, and *Sox10* prevented downstream YTHDF2-mediated mRNA decay, resulting in increased expression of these melanoma-promoting genes (Yang S. et al., 2019).

#### Readers

Few studies have examined the role of only m^6^A readers in mediating cell death. One study found that YTHDF2 negatively regulates apoptosis in TNBC (Einstein et al., 2021). Mechanistically, YTHDF2 was found to mediate the degradation of the *Prss23* mRNA transcript, a gene involved in translation, in an m^6^A-dependent manner (Einstein et al., 2021). More generally, YTHDF2-mediated mRNA decay provides a mechanism by which to control the number of translated mRNAs by degrading mRNA transcripts, therefore resulting in translational control (Einstein et al., 2021). Knockdown of *Ythdf2* subsequently lead to an increase in PRSS23 expression and protein translation, triggering ER stress and inducing proteotoxic cell death (Einstein et al., 2021). Furthermore, in chondrocytes, YTHDF1 was found to negatively regulate apoptosis by promoting the mRNA stability of anti-apoptotic *Bcl-2* in a METTL3/m^6^A-dependent manner (He et al., 2022).

While significant research efforts have been made to establish the role of RNA modifications in cell death, there remain significant gaps in knowledge surrounding this topic. Compelling evidence suggests a critical role of m^6^A RNA methylation in other forms of cell death. For example, *Guo et al.* have recently established that m^6^A may regulate macrophage pyroptosis, an inflammation-induced form of cell death, in circular RNAs in patients with acute coronary syndrome (Bergsbaken et al., 2009; Guo et al., 2020). However, the contribution of the m^6^A machinery in this cellular process remains to be investigated.

### Proliferation

The role of m^6^A in regulating cellular proliferation has been best-studied in the context of cancer. Accordingly, m^6^A writers, erasers, and readers may represent viable therapeutic targets for their role in promoting cell proliferation.

#### Writers

While the m^6^A writer METTL3 has been well-studied within the context of apoptosis, METTL3 has been found to either promote or inhibit cellular proliferation, depending on the cellular context.

METTL3 was shown to inhibit cellular proliferation in endometrial cancer, as METTL3-mediated m^6^A promoted the translation of PHLPP2, a negative regulator of pro-proliferative AKT signaling (Liu et al., 2018). Accordingly, knockdown of *Mettl3* resulted in increased cellular proliferation through decreased m^6^A-dependent translation of PHLPP2, thereby promoting AKT signaling (Liu et al., 2018). Furthermore, in renal cell carcinoma (RCC), decreased *Mettl3* expression resulted in increased proliferation through induction of the PI3K/AKT/mTOR pathway (Li X. et al., 2017). Whether the activation of the PI3K/AKT/mTOR pathway upon *Mettl3* knockdown is m^6^A-dependent was not explored (Li X. et al., 2017).

However, in several other cancer cell types, METTL3 was shown to promote cell proliferation. In colorectal cancer, METTL3 promoted GLUT1 translation in an m^6^A-dependent manner, which resulted in downstream activation of mTORC1 and increased cell survival and proliferation (Chen et al., 2021). In hepatoblastoma cells, increased METTL3 activity resulted in increased m^6^A deposition on *Ctnnb1*, leading to aberrant activation of the WNT/CTNNB1 pathway, which promoted hepatoblastoma cell growth (Liu et al., 2019). Another study in hepatocellular carcinoma (HCC) cells found that METTL3 promoted proliferation by inhibiting the expression of SOCS2, a transcription factor that can negatively regulate cell proliferation, through m^6^A-dependent/YTHDF2-mediated mRNA degradation (Chen M. et al., 2018). Furthermore, in breast cancer cells, METTL3 was found to participate in a feedback loop with HBXIP, a co-factor of anti-apoptotic protein SURVIVIN, wherein HBXIP up-regulated METTL3 expression by suppressing METTL3 inhibitor *let-7g*, an miRNA (Garcia-Saez et al., 2011; Cai et al., 2018). Increased METTL3 expression then further promoted HBXIP expression in an m^6^A-dependent manner and drove cell proliferation (Cai et al., 2018). However, whether METTL3-mediated m^6^A on *Hbxip* promotes mRNA stability or translation was not explored (Cai et al., 2018). In bladder cancer, METTL3 promoted the m^6^A-mediated maturation of pri-miR221/222, a PTEN antagonist, resulting in loss of cell cycle control and increased proliferation (Han et al., 2019). Similarly, METTL3 also drove proliferation in ovarian cancer through regulation of the receptor tyrosine kinase AXL; however, the regulatory mechanism by which METTL3 up-regulates AXL was not established (Hua et al., 2018). While the role of METTL3 in promoting proliferation in many cancer types has been well-established, the role of METTL3 in pancreatic cancer proliferation remains controversial as two independent studies found contrasting roles for METTL3 in promoting pancreatic cancer cell proliferation (Taketo et al., 2018; Xia et al., 2019). Authors of these studies reconcile differences in results based on differences in proliferation assays and cell lines used (Taketo et al., 2018; Xia et al., 2019).

Other m^6^A writer co-factors have also been found to regulate cellular proliferation in a context-dependent manner. METTL14 expression was found to be decreased in colorectal cancer patients, and *Mettl14* knockdown *in vitro* resulted in decreased m^6^A deposition on downstream target *Xist*, a long non-coding RNA (lncRNA) that has been found to promote proliferation (Yang et al., 2020). Decreased m^6^A on *Xist* prevented YTHDF2-mediated mRNA degradation, resulting in increased *Xist* expression and increased cell proliferation (Yang et al., 2020). Similarly, in gastric cancer, METTL14 decreased cell proliferation by negatively regulating the pro-proliferative PI3K/AKT/mTOR pathway, emphasizing the cell-type-specific role of METTL14 in this cellular process (Liu X. et al., 2021). Whether METTL14 regulates the PI3K/AKT/mTOR pathway in an m^6^A-dependent manner remains unclear (Liu X. et al., 2021). METTL14 can also promote cell proliferation. In breast cancer, METTL14 was recruited by oncogenic lncRNA *LINC00942* to increase the m^6^A-mediated mRNA stability and protein expression of two downstream targets, CXCR4 and CYP1B1, which resulted in increased cell proliferation and tumorigenesis (Sun et al., 2020). In AML, METTL14 promoted cell survival and proliferation by regulating the mRNA stability and translation of two pro-proliferative downstream targets, MYB and MYC, in an m^6^A-dependent manner (Weng et al., 2018). In skin cancer, METTL14 was also found to promote cell proliferation, as knockdown of *Mettl14* in human keratinocytes resulted in decreased cell proliferation; however, the mechanism by which METTL14 promotes proliferation in this context remains unclear (Yang Z. et al., 2021).

WTAP, another m^6^A writer co-factor, has been found to promote proliferation. However, studies examining the role of WTAP in regulating proliferation do not detail whether WTAP promotes proliferation in an m^6^A-dependent manner. In renal cell carcinoma (RCC), WTAP promoted the mRNA stability of *Cdk2*, a regulator of cell cycle control over the G1/S and S/G2 transition, by directly binding to the *Cdk2* transcript at the 3′-UTR (Tang et al., 2018). Furthermore, in primary AML patient samples and AML cell lines, reverse phase protein array (RPPA) analysis revealed that WTAP is positively associated with pro-proliferative cyclins and HSP90, as well as anti-apoptotic proteins, such as BCL-2 (Bansal et al., 2014). The mechanism by which WTAP regulates the expression of these pro-proliferative proteins, and whether this regulation is m^6^A-dependent, was not explored in this study (Bansal et al., 2014).

Additionally, VIRMA was found to promote non-small cell lung cancer (NSCLC) and increased NSCLC proliferation *in vitro* and *in vivo* (Xu et al., 2021). Mechanistically, VIRMA promoted the mRNA decay of tumor suppressor *Dapk3* through an m^6^A-dependent YTHDF2/YTHDF3-mediated mechanism (Xu et al., 2021). Furthermore, VIRMA was found to promote breast cancer progression by promoting the mRNA stability of *Cdk1* in an m^6^A-independent manner (Qian et al., 2019).

#### Erasers

The pro-proliferative role of the m^6^A eraser FTO is well-studied within the context of cancer. In leukemia, FTO promoted the proliferation of AML cells by reducing m^6^A levels at the 3′-UTR of *Asb2* and 3′ and 5′-UTR of *Rara*, two mediators of hematopoiesis and differentiation, resulting in decreased ASB2 and RARA protein expression (Li Z. et al., 2017). In melanoma, FTO promoted cell proliferation and overall tumorigenicity by demethylating m^6^A on melanoma-promoting genes *Pdcd1*, *Cxcr4*, and *Sox10* (Yang S. et al., 2019). Furthermore, exposure to arsenic, a known human carcinogen, resulted in increased FTO stability and abundance in human keratinocytes, ultimately leading to increased proliferation and tumorigenesis (Cui et al., 2021). Furthermore, FTO and MYC have also been found to cooperate to drive cell proliferation in both pancreatic and cervical cancer (Tang et al., 2019; Zou et al., 2019). In pancreatic cancer, FTO mediated the m^6^A demethylation of the *c-Myc* transcript, resulting in increased c-MYC expression (Tang et al., 2019). In cervical cancer, FTO was found to promote MYC translation; however, whether this mechanism was m^6^A-dependent was not established (Zou et al., 2019). FTO also promoted the proliferation of NSCLC cells by demethylating and increasing the mRNA stability of the ubiquitinase *Usp7*, resulting in increased USP7 protein expression (Li et al., 2019). Future studies are needed to define the role of USP7 in mediating cell proliferation (Li et al., 2019).

In addition to FTO, m^6^A eraser ALKBH5 drove proliferation in glioblastoma stem cells by demethylating nascent mRNA transcripts of *Foxm1*, a transcription factor involved in cell-cycle control and proliferation, resulting in increased FOXM1 expression and activity in an m^6^A-dependent manner (Zona et al., 2014; Zhang S. et al., 2017).

#### Readers

The m^6^A reader YTHDF2 promotes cell proliferation across different cell types and through distinct mechanisms. In pancreatic cancer, YTHDF2 promoted cell growth through activation of the AKT/GSK3β/CCND1 pathway (Chen et al., 2017). However, it is unclear whether YTHDF2 mediates pancreatic cancer growth in an m^6^A-dependent manner (Chen et al., 2017). In leukemia, YTHDF2 increased cell proliferation by promoting the m^6^A-dependent mRNA decay of *Wee1*, which regulates mitotic entry and serves as a negative cell-cycle regulator (Fei et al., 2020).

Additionally, PRRC2A, an m^6^A-binding protein, promoted the proliferation of oligodendrocytes, a class of glial cells found in the brain and central nervous system, by binding and stabilizing the *Olig2* mRNA transcript in an m^6^A-dependent manner (Wu et al., 2019).

While the role of m^6^A in proliferation is widely studied in the context of cancer, cell proliferation is critical for other biological processes, such as wound repair and development, and is dysregulated in many diseases. Future studies are needed to address the role of m^6^A in proliferation in these contexts.

### Senescence

Senescence is a cellular mechanism wherein cells permanently undergo cell cycle arrest in response to cellular stress or other stimuli (Kumari and Jat, 2021). Intracellularly, senescent cells undergo metabolic and genomic changes that promote cell survival, yet in a growth-arrested state (Kumari and Jat, 2021). Extracellularly, senescent cells communicate with neighboring cells through a variety of secreted factors, including cytokines and chemokines, and assume a senescence-associated secretory phenotype (SASP) (Kumari and Jat, 2021). The role of m^6^A in senescence has been studied in a variety of contexts, including tumorigenesis and aging, and is reviewed in detail elsewhere (Casella et al., 2019). In this section, we will summarize recent advances on the role of m^6^A in senescence.

#### Writers

*Liu* et al. established that the m^6^A writers METTL3 and METTL14 promoted SASP in lung embryonic fibroblasts in an m^6^A-independent manner (Liu P. et al., 2021). During cellular senescence, METTL14 was found to localize to enhancer subunits, while METTL3 localized to promoters of SASP genes (Liu P. et al., 2021). Interestingly, WTAP was found to be required for the nuclear localization of METTL3 and METTL14 during senescence (Liu P. et al., 2021). However, METTL3 may function to inhibit senescence in human mesenchymal stem cells (hMSCs), as knockdown of *Mettl3* in hMSCs resulted in accelerated senescence (Wu et al., 2020). Overexpression of *Mettl3* in hMSCs reversed the phenotype seen in *Mettl3*-deficient hMSCs and delayed senescence induction through m^6^A/IGF2BP2-mediated stabilization of the pro-proliferative gene *Mis12* (Wu et al., 2020). Furthermore, in human nucleus pulposus cells, METTL14 positively regulated TNFα-induced cellular senescence by promoting the maturation of *miR-34a-5p*, which inhibits SIRT1, a negative regulator of senescence (Zhu H. et al., 2021). However, the role of *miR-34a-5p* in senescence is not completely understood.

#### Erasers

The m^6^A eraser FTO has been found to serve as a negative regulator of senescence in various contexts. Accordingly, FTO negatively regulated cellular senescence in granulosa-cell-induced ovarian aging in an m^6^A-dependent manner (Jiang et al., 2021). In this context, expression of catalytically inactive mutant FTO, which lacks demethylase activity, increased m^6^A on the 3′-UTR of *Fos*, a transcription factor that promotes aging, preventing the m^6^A-mediated degradation of *Fos* mRNA and increasing FOS translation (Jiang et al., 2021). Similar deactivating mutations in FTO resulted in increased senescence in skin fibroblasts (Boissel et al., 2009). While the mechanism by which FTO inhibits senescence was not delineated, these studies suggest that the demethylase activity of FTO is required to inhibit senescence (Boissel et al., 2009; Jiang et al., 2021).

#### Readers

The role of readers in cellular senescence is not well-studied. In human ovarian epithelial cells, RAS activation resulted in increases in Reactive Oxygen Species (ROS), which led to decreased expression of YTHDF2 (Zhu et al., 2020). Decreased expression of YTHDF2, which functions to mediate mRNA decay, resulted in downstream activation of the MAPK pathway and prevented the mRNA decay of *Map2k4* and *Map4k4* (Zhu et al., 2020). Activation of the MAPK pathway then led to downstream activation of NF-κB signaling pathways, resulting in the induction of SASP and senescence (Zhu et al., 2020).

A current gap in knowledge in this field revolves around our understanding of the m^6^A machinery in cell fate decisions. Future studies should be centered on understanding the dynamic nature of m^6^A in initiating cellular senescence and quiescence, as well as the changes in m^6^A that are needed for the cell to re-enter the cell cycle. Elucidating the roles of m^6^A machinery in cell fate decisions has broad-standing implications in understanding stem cell biology, cancer stem cell formation and maintenance, and cell cycle control.

### Differentiation

Cell differentiation is the process of transformation into specialized cell types and is essential for development. The hematopoietic system is a well-established model which emphasizes the cell-type and stage-specific role of m^6^A in differentiation. Outside of hematopoiesis, m^6^A has been found to be a critical regulator in stem cell fate, neuronal development, and skin development. The role of m^6^A in development and stem cell biology is reviewed extensively elsewhere (Frye et al., 2018; Malla et al., 2019; Rosselló-Tortella et al., 2020; Vasic et al., 2020; Song et al., 2021). Here we will summarize the role of m^6^A in differentiation in several contexts.

#### Writers

The role of RNA modifications within hematopoietic differentiation is stage-specific. At early stages, m^6^A is necessary for differentiation during the endothelial to hematopoietic transition (EHT), which mediates early-stage hematopoietic stem and progenitor cell (HSPC) differentiation (Zhang C. et al., 2017). The necessity of m^6^A within EHT is demonstrated by *mettl3* ^−/−^ zebrafish, which display disrupted HSPC development (Zhang C. et al., 2017). Mechanistically, *mettl3* ^−/−^ zebrafish show continuous Notch activation, as depletion of m^6^A on the *notch1a* transcript prevents Ythdf2-mediated *notch1a* mRNA decay (Zhang C. et al., 2017). Continuous Notch activation in *mettl3* ^−/−^ zebrafish promotes an endothelial cell lineage, thereby inhibiting EHT and preventing the HPSC generation (Zhang C. et al., 2017). In mice, conditional *Mettl3* knockout promoted hematopoietic stem cell (HSC) accumulation in the bone marrow, suggesting that HSC differentiation was unable to progress without METTL3 or m^6^A (Lee et al., 2019). Mechanistically, METTL3-mediated m^6^A is believed to promote the mRNA translation of downstream target *Myc*, which regulates differentiation; *Mettl3* ^−/−^ mice therefore display a differentiation block due to decreased MYC translation (Lee et al., 2019). Other independent studies have also noted blocks in HSC differentiation in *Mettl3* ^−/−^ mice, establishing a pervasive role for METTL3-mediated m^6^A within differentiation (Cheng et al., 2019). However, knockdown of *Mettl3* in HSPCs resulted in increased cellular differentiation, emphasizing the stage-specific function of m^6^A within differentiation (Vu et al., 2017). Furthermore, METTL3-mediated m^6^A was found to inhibit differentiation in AML cells, which suggests that m^6^A may have distinct functions upon oncogenic transformation in AML cell lines (Lee et al., 2019).

In embryonic stem cells, m^6^A was found to be critical for mediating the mRNA decay and turnover of transcripts within differentiation (Batista et al., 2014). Similarly, in the context of neuronal development, *Yoon* et al. identified m^6^A to be a critical factor in mediating neurogenesis, as m^6^A was found to promote the mRNA decay of transcription factors involved in this process (Yoon et al., 2017). m^6^A was also found to regulate embryonic neural stem cell renewal and differentiation through regulation of histone modifications, which may further influence the transcription or expression of transcription factors involved in neuronal development (Wang Y. et al., 2018). PRRC2A, an m^6^A-binding protein, also promoted the fate determination of oligodendrocytes through stabilization of the *Olig2* mRNA transcript in an m^6^A-dependent manner (Wu et al., 2019). Together, these studies establish the critical role of m^6^A in mediating the coordination in gene expression events in stem cell differentiation.

Differentiation is a key process in skin development, homeostasis, and wound repair (Lopez-Pajares et al., 2013). Accordingly, *Lee* et al. determined that METTL14-dependent m^6^A methylation on lncRNA *Pvt1* regulates stemness in epidermal progenitor cells, promoting both *Pvt1*-MYC interactions and MYC protein stabilization (Lee J. et al., 2021).

Furthermore, WTAP was found to be an essential factor for mediating the differentiation of endoderm and mesoderm as mouse embryos lacking *Wtap* failed to differentiate into endoderm and mesoderm and were embryonic lethal during the gastrulation phase of development (Fukusumi et al., 2008). *Horiuchi* et al. also found that loss of *Wtap* resulted in embryonic lethality at day 6.5 (Horiuchi et al., 2006). Mechanistically, this study found that WTAP promoted the stabilization of *Cyclin A2* mRNA, which regulates the G_2_/M transition, and that loss of *Wtap* resulted in G_2_ accumulation and subsequent lethality (Horiuchi et al., 2006).

#### Erasers

The m^6^A eraser FTO may regulate differentiation across different cell types. However, the role of FTO in promoting, or inhibiting, differentiation is cell-type dependent. Knockout of *Fto* in adult neural stem cells (aNSCs) resulted in increased aNSC proliferation and differentiation through aberrant activation of the STAT3 pathway, resulting in inhibited neurogenesis and dysregulated neuronal development (Cao et al., 2019). Mechanistically, the STAT3 pathway was activated through increased m^6^A enrichment on *Pdgfrα* and *Socs5* mRNA transcripts, due to decreased FTO expression and activity (Cao et al., 2019). Interestingly, decreased FTO expression and activity resulted in increased PDGFRα protein expression and decreased SOCS5 protein expression, which, together, promote the phosphorylation and activation of STAT3 (Cao et al., 2019). The differences between m^6^A-dependent regulation of PDGFR*α* and SOCS5 protein expression were not explored in this study (Cao et al., 2019).

FTO is also involved in adipogenic differentiation. Accordingly, decreased FTO demethylase activity resulted in decreased preadipocyte differentiation in an m^6^A-dependent manner, and FTO-over-expressing mouse embryonic fibroblasts (MEFs) showed increased adipogenic differentiation (Merkestein et al., 2015; Zhang et al., 2015).

#### Readers

The m^6^A reader YTHDF2 was identified to function as the main regulator of mRNA decay of transcriptional regulators involved in hematopoiesis and self-renewal (Li et al., 2018). *Ythdf2* ^−/−^ HSPCs resulted in increased expansion of HSCs and increased mRNA expression of transcription factors involved in self-renewal, such as *Gata2*, *Runx1*, *Tal1*, and *Stat5* (Li et al., 2018). Mechanistically, YTHDF2 is believed to negatively regulate HSC expansion by facilitating the mRNA decay of *Gata2*, *Runx1*, *Tal1*, and *Stat5* in an m^6^A-dependent manner (Li et al., 2018). Furthermore, in *mettl3* ^−/−^ zebrafish, decreased m^6^A resulted in decreased Ythdf2-mediated mRNA decay of *notch1a*, a transcription factor that represses HSPC formation (Zhang C. et al., 2017).

YTHDC1 also serves a role in differentiation as *Ythdc1* expression was increased in M0 undifferentiated acute myeloblastic leukemia cells, suggesting that YTHDC1 may be required to maintain an undifferentiated state (Cheng et al., 2021). Furthermore, knockdown of *Ythdc1* in the OCIAML3 cell line resulted in increased differentiation (Cheng et al., 2021). Mechanistically, YTHDC1 is believed to inhibit differentiation through downstream m^6^A-dependent regulation of MYC (Cheng et al., 2021).

m^6^A plays crucial roles in differentiation in the hematopoietic system, as well as in stem cell fate, neuronal development, and skin development. Expanded studies should be employed to specifically address the dynamic changes in m^6^A machinery across totipotent, multipotent, and pluripotent stem cells.

### Migration

Cell migration involves the coordination of biophysical and mechanical mechanisms that allow cells to migrate. Cell migration is also the major cellular process that drives wound healing, cancer progression, and metastasis.

#### Writers

METTL14 is the best-studied m^6^A writer in the context of migration. Many studies have found that METTL14 may serve as either a positive or negative regulator of migration and metastasis, depending on cellular context. In gastric cancer and endometrial cancer, knockdown of *Mettl14* and decreased m^6^A levels increased cell migration and invasiveness, establishing METTL14 as a negative regulator of migration and metastasis in these contexts (Liu et al., 2018; Zhang C. et al., 2019). Furthermore, in colorectal cancer, METTL14 inhibited migration and metastasis through m^6^A/YTHDF2-mediated mRNA degradation of the epithelial-to-mesenchymal transition (EMT)-promoting transcription factor *Sox4* (Chen X. et al., 2020). Furthermore, in papillary thyroid cancer, METTL14 inhibited migration by binding, and decreasing the expression of, lncRNA *OIP5-As1*, which promotes proliferation and migration through downstream regulation of the MEK/ERK, EGFR, and PI3K pathways (Zhang et al., 2021). However, it is unclear whether this mechanism is m^6^A-dependent (Zhang et al., 2021). Conversely, in keratinocytes and skin cancer cells, METTL14 promoted migration in an m^6^A dependent manner, as knockdown of *Mettl14* decreased migration, while overexpression of wild-type, but not catalytically inactive mutant, *Mettl14* resulted in increased migration (Yang Z. et al., 2021).

Similarly, METTL3 may promote or inhibit migration depending on the cellular context. Several studies in melanoma have found that METTL3 induced migration by increasing expression of pro-migratory proteins, c-MET and MMP2, in an m^6^A-dependent manner (Spina et al., 2015; Dahal et al., 2019; Luo et al., 2020). In lung cancer, METTL3 was increased during TGF-β-induced EMT (Wanna-Udom et al., 2020). Furthermore, in liver cancer, METTL3 was found to mediate increases in m^6^A levels during EMT, including specific m^6^A increases on the coding sequence (CDS) of EMT-associated transcription factor *Snail*, resulting in YTHDF1-mediated increases in SNAIL translation and EMT progression (Lin X. et al., 2019). Similarly, in bladder cancer, METTL3 deposited m^6^A on the 3′-UTR of *Cdcp1*, which has been found to promote migration across several cancer types, resulting in YTHDF1-mediated increases in CDCP1 translation and increased cellular migration (Yang F. et al., 2019). In NSCLC and gastric cancer, METTL3 promoted migration through downstream activation of PI3K/AKT; however, whether this mechanism is m^6^A-dependent is unclear (Lin S. et al., 2019; Wei et al., 2019). Interestingly, METTL3 expression in ovarian cancer also increased migration and induction of EMT through increased protein expression of the receptor tyrosine kinase AXL; however, while the *Axl* mRNA transcript contains fourteen m^6^A sites, METTL3 regulation of AXL translation is believed to be m^6^A-independent (Hua et al., 2018). The m^6^A-independent mechanism by which METTL3 regulates AXL translation remains unclear (Hua et al., 2018). In contrast, in colorectal cancer cells, *Mettl3* over-expression resulted in decreased migration, while decreased *Mettl3* activated the p38/ERK pathways, resulting in increased migration (Deng et al., 2019). Whether regulation of p38/ERK by METTL3 is m^6^A-dependent was not explored in this study (Deng et al., 2019).

Another m^6^A writer-associated protein, WTAP, also induced migration and metastasis by increasing the mRNA expression of migration-promoting genes, *Mmp7*, *Mmp28*, *Cathepsin H*, and *Muc1* in cholangiocarcinoma cells (Jo et al., 2013). However, this study did not investigate whether WTAP-mediated increases in *Mmp7*, *Mmp28*, *Cathepsin H*, and *Muc1* are m^6^A-dependent (Jo et al., 2013).

#### Erasers

The role of the m^6^A eraser FTO in migration is not well-established and requires further study. A study in cervical cancer suggested that FTO regulates migration by promoting the protein translation of E2F1 and MYC, two regulators of cell cycle and migration, in an m^6^A-dependent manner (Zou et al., 2019). Furthermore, in melanoma cells, overexpression of FTO promoted migration and overall tumorigenicity in an m^6^A-dependent manner, while knockdown of FTO inhibited migration (Yang S. et al., 2019).

#### Readers

The m^6^A reader YTHDF2 may have inhibitory effects on migration. m^6^A deposition on lncRNA THOR contributes to increased migration across many different cancer cell types (Liu H. et al., 2020). Interestingly, m^6^A on THOR is read by YTHDF1 and YTHDF2, which can mediate the transcription or decay of THOR, respectively, and therefore influence migration through their respective effects on THOR RNA metabolism (Liu H. et al., 2020). In pancreatic cancer, YTHDF2 was involved in a “migration-proliferation dichotomy” wherein YTHDF2 promoted proliferation but inhibited migration by suppressing YAP signaling, an EMT-promoting signaling pathway (Chen et al., 2017). While YAP contains two m^6^A sites, it is unclear whether YTHDF2 regulates YAP expression by directly regulating mRNA stability, or whether YTHDF2 regulates upstream regulators of YAP (Chen et al., 2017).

While many studies have reported the effects of RNA modification by writers, erasers and readers on regulating migration, the unique biophysical mechanisms that underlie these transitions are not well-elucidated and remain an active area of research. For example, future studies are needed to explore the potential role of RNA modifications in regulating cytoskeletal proteins.

### Metabolism

m^6^A mediates cellular metabolism in a cell-type dependent manner. The intersection of epitranscriptomics and metabolism remains an understudied area of research. The role of m^6^A in mediating cancer metabolism is further reviewed elsewhere (Han et al., 2020).

#### Writers

The m^6^A writer METTL3 promotes lipogenesis and adipogenesis across several different contexts. In HCC cell lines, METTL3-mediated m^6^A promoted lncRNA *LINC00958* RNA stability in an m^6^A-dependent manner (Zuo et al., 2020). With increased RNA stability, *LINC00958* promoted lipogenesis by regulating the *miR-3619-5p*/HDGF pathway, which, in turns, regulates lipogenesis enzymes such as SREB1, FASN, SCD1, and ACC1 (Zuo et al., 2020). As a result, increased *LINC0095* RNA stability resulted in increased cholesterol and triglyceride levels and lipid droplet formation (Zuo et al., 2020). Interestingly, FTO and METTL3 may communicate to coordinate adipogenesis and fat absorption, as visceral fat taken from offspring of high-fat diet-fed mice mothers exhibited decreased FTO expression, and increased METTL3 expression, at 3 weeks of age (Li et al., 2016). Increased m^6^A levels were also noted at 3 weeks of age in the visceral fat of these offspring (Li et al., 2016). However, at 8 weeks of age, both FTO and METTL3 were increased in the visceral fat, despite their contradictory functions, with no changes in m^6^A levels noted (Li et al., 2016). These results suggest a unique coordination between FTO and METTL3 in response to a high-fat diet and within development, but the mechanism remains unclear (Li et al., 2016). In addition to lipogenesis and adipogenesis, METTL3 was found to regulate glucose metabolism in colorectal cancer, as METTL3-mediated m^6^A promoted the mRNA stability of *Hk2* and *Glut1* in an m^6^A and IGF2BP2/3-dependent manner (Shen et al., 2020). Increased *Hk2* and *Glut1* mRNA stability subsequently led to the activation of glycolysis and promoted colorectal cancer cell progression (Shen et al., 2020).

#### Erasers

FTO serves a pivotal role in multiple metabolic processes, including fat metabolism, gluconeogenesis, metabolic stress, and lactate production. Several seminal studies were intrinsic to establishing the role of FTO in fat metabolism. However, because FTO was only recently established as an m^6^A eraser, the contexts in which FTO promotes obesity in an m^6^A-independent or dependent manner is not entirely clear. In humans, the FTO SNP rs9939609 was found to be linked to body mass index and is believed to be one of the strongest genetic determinants of obesity propensity (Frayling et al., 2007). Additionally, the FTO SNP rs8050136 in humans decreases the binding affinity of the CUX1 isoform P110, resulting in decreased expression of FTO and leptin signaling, preventing satiety, and promoting obesity (Stratigopoulos et al., 2011).

**FIGURE 3:**
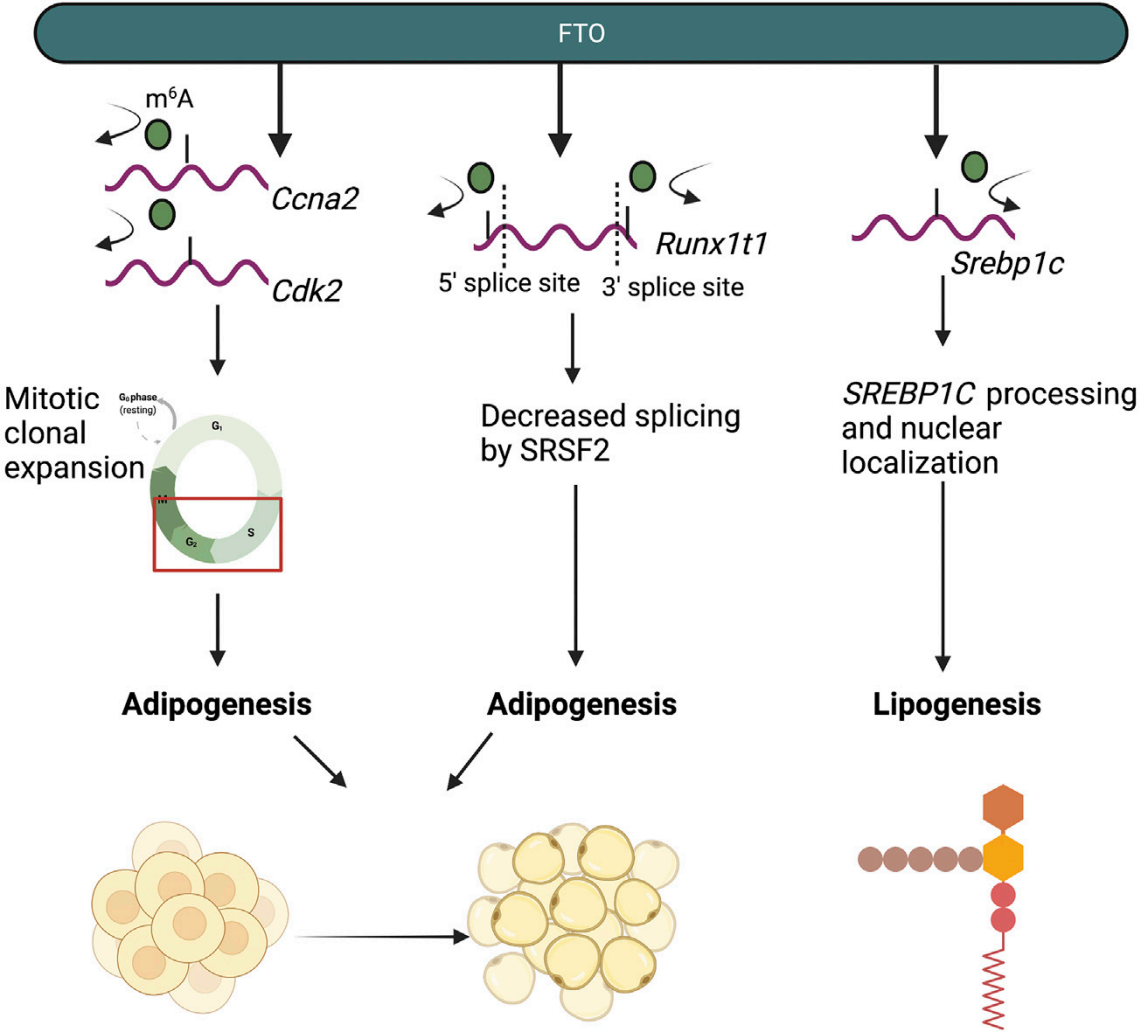
The m^6^A-dependent roles of FTO in adipocyte and lipid metabolism. FTO regulates and promotes adipogenesis and lipogenesis by demethylating mRNA transcripts of genes involved in adipogenesis (*Ccna2*, *Cdk2*, *Runx1t1*) and lipogenesis (*Srebp1c*).

More recent studies have suggested that the demethylase activity of FTO is indispensable for its role in mediating fat metabolism, including lipogenesis and adipogenesis. Decreased m^6^A levels, mediated by the demethylase activity of FTO, were found to increase triglyceride deposition in HepG2 hepatocyte cells (Kang et al., 2018). Additional studies in HepG2 cells show that FTO mediated an increase in the expression of SREBP1c and CIDEC, two transcriptional regulators of lipogenesis, by increasing their nuclear localization, thereby promoting lipogenesis (Wang Y. et al., 2015; Chen A. et al., 2018). Increases in SREBP1c protein expression were found to be m^6^A-dependent, as mutant FTO was did not mediate changes in SREBP1c processing or protein expression (Chen A. et al., 2018). The mechanism by which FTO-mediated m^6^A demethylation mediates CIDEC expression remains unclear (Chen A. et al., 2018). In 3T3-L1 preadipocytes, FTO promoted adipogenesis through regulation of cell cycle progression (Wu et al., 2018). Mitotic clonal expansion (MCE) is a pivotal prerequisite process required for adipocyte differentiation and adipogenesis (Tang et al., 2003). Within this process, differentiating adipocytes are required to enter the cell cycle and proliferate (Tang et al., 2003). In 3T3-L1 cells with *Fto* knockdown, increased m^6^A levels resulted in decreased mRNA expression of cell-cycle control genes *Ccna2* and *Cdk2*, which regulate the S to G2 transition (Wu et al., 2018). Subsequently, increased m^6^A levels on *Ccna2* and *Cdk2* mRNA transcripts resulted in m^6^A-dependent/YTHDF2-mediated decay of *Ccna2* and *Cdk2* mRNA and decreased CCNA2 and CDK2 protein expression (Wu et al., 2018). Decreased expression of CCNA2 and CDK2 resulted in the impairment of MCE and preadipocytes were unable to progress to the G2 phase, halting preadipocyte development (Wu et al., 2018). FTO has also been found to regulate RUNX1T1, an adipocyte transcription factor (Zhao et al., 2014). Within the *Runx1t1* mRNA transcript, m^6^A was enriched at exonic regions near 5′ and 3′ splice sites; accordingly, increased m^6^A levels, mediated through *Fto* knockdown, led to increased binding by the splicing protein SRSF2, which resulted in changes in exon splicing and inclusion in the *Runx1t1* transcript (Zhao et al., 2014; Zhang B. et al., 2020). The m^6^A-dependent roles of FTO in lipid and adipocyte metabolism are highlighted in Figure 3.

FTO may also serve a role in regulating gluconeogenesis. Increased glucose uptake induced the expression of *Fto*, resulting in overall decreases in m^6^A (Yang Y. et al., 2019). High FTO expression was also correlated with increased mRNA expression of genes involved in glucose and lipid metabolism, including *Foxo1*, *G6pc*, *Dgat2*, and *Fasn*, upon glucose stimulation (Jensen-Urstad and Semenkovich, 2012; Kousteni, 2012; Yang Y. et al., 2019). However, the mechanism by which FTO regulates the expression of *Foxo1*, *G6pc*, *Dgat2*, and *Fasn* was not explored in this study (Yang Y. et al., 2019). In another study, FTO was found to demethylate m^6^A sites on *Foxo1*, resulting in increased FOXO1 expression, and increased gluconeogenesis (Peng et al., 2019). Interestingly, *Foxo1* mRNA expression was not changed by changes in FTO expression or activity (Peng et al., 2019). Rather, mutating an internal m^6^A site on the *Foxo1* mRNA transcript prevented FTO-mediated increases in FOXO1, establishing that the internal m^6^A site on the *Foxo1* mRNA transcript is required for the FTO-FOXO1 axis (Peng et al., 2019).

Furthermore, FTO and ALKBH5 may regulate metabolism in response to cellular stress through interactions with ATF4, a stress-response gene and major regulator of cellular metabolism. Under stress conditions, ATF4 expression increases (Zhou et al., 2018). However, upon *Fto* or *Alkbh5* knockdown in MEF and 293T cells, ATF4 expression failed to increase upon amino acid starvation (Zhou et al., 2018). Mechanistically, m^6^A methylation on the 5′-UTR of *Atf4* is dynamically changed in response to stress; increased m^6^A methylation on the *Atf4* mRNA transcript due to *Fto* or *Alkbh5* knockdown results in decreased ATF4 translation in response to amino acid starvation (Zhou et al., 2018). Conversely, *Mettl3* or *Mettl14* knockdown in this context resulted in increased ATF4 translation upon amino acid starvation, suggesting that the ATF4 regulation in response to starvation is m^6^A-dependent (Zhou et al., 2018).

Additionally, ALKBH5 was found to regulate lactate production in melanoma and colon cancer (Li et al., 2020). Knockdown of *Alkbh5* in melanoma and colon cancer cells resulted in m^6^A-dependent decreases in the stability of *Mct4/Slc16a3* mRNA, a regulator of lactate secretion (Li et al., 2020). Accordingly, *Alkbh5* knockdown resulted in decreased lactate production in the tumor interstitial fluid of the tumor microenvironment in both melanoma and colon cancer (Li et al., 2020).

#### Readers

The role of readers in mediating metabolism requires future study. However, one study showed that m^6^A on mitochondrial carrier homology 2 (*Mtch2*) mRNA resulted in increased MTCH2 protein expression, resulting in increased adipogenesis in longissimus dorsi muscle cells taken from both lean Landrace-breed pigs and obese Jinhua-breed pigs (Jiang et al., 2019).

The role of m^6^A in metabolism is an exciting new field of interest. Few studies have addressed the role of other MTC proteins, such as WTAP and VIRMA, in regulating metabolism. Other potential directions the field could address include how the cellular microenvironment influences cell-intrinsic changes in m^6^A and how changes in m^6^A can contribute to changes in cancer cell metabolism.

### Autophagy

Autophagy is a conserved cellular process that is mobilized during the stress response and within normal housekeeping functions. The autophagic process involves the removal and degradation of excessive or damaged organelles or proteins, as well as other biological molecules, into membrane-bound autophagosomes (Klionsky, 2007; Mizushima et al., 2008). m^6^A has been found to regulate autophagy; however, the discrete mechanisms and cellular contexts with which m^6^A influence autophagy remain unexplored (Frankel et al., 2017; Abildgaard et al., 2020).

#### Writers

The m^6^A writer METTL3 has been found to negatively regulate autophagy across several contexts. In HCC, METTL3 inhibited autophagy by depositing m^6^A at the 3′-UTR of *Foxo3a*, a negative regulator of autophagy (Lin et al., 2020). m^6^A on the 3′-UTR of *Foxo3a* resulted in downstream YTHDF1-mediated *Foxo3a* mRNA stabilization and subsequently inhibited autophagy (Lin et al., 2020). Furthermore, in an ischemic heart model, METTL3-mediated m^6^A on the 3′-UTR of *Tfeb*, which promotes autophagy and lysosome biogenesis, resulted in HNRNPD-mediated decreases in *Tfeb* mRNA expression and decreased autophagy (Song et al., 2019).

Autophagy can also promote therapeutic resistance and cell survival. Accordingly, METTL3 promoted resistance to gefitinib in NSCLC through regulation of two core autophagy genes, *Atg5* and *Atg7* (Glick et al., 2010; Liu S. et al., 2020). Accordingly, *Mettl3* knockdown in NSCLC cells resulted in decreased *Atg5* and *Atg7* mRNA expression (Liu S. et al., 2020). However, whether this regulation was m^6^A-dependent is unclear (Liu S. et al., 2020).

Additionally, in human keratinocytes, METTL14 abundance was found to be down-regulated by UVB exposure through NBR1-mediated selective autophagy (Yang Z. et al., 2021). Furthermore, mTORC1, a negative regulator of autophagy, promoted the stabilization of the MTC consisting of METTL3, METTL14, WTAP, and RMB15/RBM15B (Tang et al., 2021). Mechanistically, mTORC1 promoted the stabilization of the MTC by regulating the chaperonin CCT, which facilitates protein folding and stabilization of the MTC in *Drosophila* S2R+ and human HEK293T cells (Tang et al., 2021). The mechanism by which mTORC1 regulates CCT is detailed further elsewhere (Tang et al., 2021). Increased stabilization of the MTC led to increased m^6^A deposition and mRNA degradation of two downstream targets, autophagy genes *Atg1* and *Atg8a*, resulting in the suppression of autophagy (Tang et al., 2021).

Furthermore, one study found that WTAP could regulate autophagy by mediating the phosphorylation of the positive autophagy regulator, AMPK, in HCC cells (Li G. et al., 2021). Mechanistically, WTAP decreased the mRNA stability of *Lkb1*, the kinase upstream of AMPK which regulates AMPK phosphorylation, in an m^6^A-dependent manner (Li G. et al., 2021). Subsequently, knockdown of *Wtap* resulted in increased autophagy (Li G. et al., 2021).

#### Erasers

The role of the m^6^A eraser FTO as a regulator of autophagy has been studied in a variety of different contexts. In HeLa cells, knockdown of *Fto* decreased autophagic flux (Jin et al., 2018). Interestingly, only the catalytically active form of FTO was able to increase autophagic flux, evidenced by increased LC3B puncta in cells expressing wild-type, but not catalytically inactive mutant, FTO, which suggests that FTO regulates autophagy in an m^6^A-dependent manner (Jin et al., 2018). RNA immunoprecipitation (RIP)-qPCR revealed that FTO binds to *Ulk1* mRNA, a gene involved in the initial stages of autophagy and is an important recruitment factor in autophagosome formation (Zachari and Ganley, 2017; Jin et al., 2018). The interaction between FTO and *Ulk1* was further elucidated as three m^6^A sites were found in the 3′-UTR of the *Ulk1* transcript, which were subsequently targeted for degradation by YTHDF2 (Jin et al., 2018). FTO-mediated demethylation of *Ulk1* may therefore preserve *Ulk1* from YTHDF2-mediated degradation (Jin et al., 2018). In addition to ULK1, FTO may also preserve core autophagy genes, *Atg5* and *Atg7*, from YTHDF2-mediated degradation in adipocytes (Glick et al., 2010; Wang et al., 2020). Knockdown of *Fto* in 3T3-L1 cells increased m^6^A levels across the *Atg5* and *Atg7* mRNA transcripts, resulting in YTHDF2-mediated degradation and inhibition of autophagy (Wang et al., 2020). Interestingly, knockdown of *Fto* in 3T3-L1 cells did not change m^6^A levels on ULK1, emphasizing the cell-type dependent regulation of autophagy by FTO (Wang et al., 2020).

FTO has also been shown to be a target for p62-dependent selective autophagy. In human keratinocytes, FTO protein expression was stabilized and up-regulated by arsenic exposure through inhibition of p62-mediated autophagy (Cui et al., 2021).

Furthermore, the m^6^A eraser ALKBH5 was found to promote autophagy in Leydig cells (Chen Y. et al., 2020). Mechanistically, m^6^A promoted the translation of PPM1A, a negative AMPK regulator, in a YTHDF1-dependent manner (Chen Y. et al., 2020). Furthermore, m^6^A also reduced the mRNA stability of *Camkk2*, a positive AMPK regulator, in a YTHDF2-dependent manner, resulting in autophagy inhibition (Chen Y. et al., 2020). However, decreased m^6^A methylation, mediated by ALKBH5, resulted in autophagy induction by preventing *Camkk2* mRNA decay (Chen Y. et al., 2020).

#### Readers

Few studies have examined the role of only m^6^A readers in autophagy. YTHDC1 was found to regulate autophagy in human keratinocytes treated with high glucose, as knockdown of *Ythdc1* resulted in decreased autophagic flux (Liang et al., 2021). Mechanistically, YTHDC1 promoted mRNA stability of the autophagy receptor *Sqstm1* in an m^6^A-dependent manner (Liang et al., 2021). Accordingly, knockdown of *Ythdc1* resulted in *Sqstm1* mRNA degradation, leading to decreased autophagic flux (Liang et al., 2021). Furthermore, in HCC, YTHDF1 was identified to positively regulate autophagy by promoting the translation of core autophagy proteins ATG2A and ATG14 under hypoxic conditions in an m^6^A-dependent manner (Li Q. et al., 2021).

As this is a relatively new field of research, future studies are needed to identify the context-dependent role of m^6^A at different stages in the autophagic process, from the formation of phagophore, autophagosome, and autolysosome, to cargo degradation in the lysosomes, and identify the different cellular stressors and stimuli that mediate dynamic m^6^A changes within this process (Glick et al., 2010).

### DNA Damage Response

Elucidating the communication between m^6^A and DNA damage response (DDR) is an active area of study. The role of m^6^A in modulating these pathways will add new insights into the DDR machinery.

#### Writers

In response to UVC or UVA radiation, m^6^A and DNA Pol κ were rapidly recruited to sites of DNA damage (Xiang et al., 2017). While the detailed mechanism behind the role of DNA Pol κ in DDR is not fully understood, the catalytic activity of METTL3 was found to be required for DNA Pol κ recruitment to DNA damage sites (Xiang et al., 2017). Knockout of *Mettl3* in HeLa and U2OS cells exposed to UV radiation resulted in decreased cyclobutene pyrimidine dimer (CPD) removal, a major UV damage product, (Xiang et al., 2017). Knockout of *Mettl14* in human keratinocyte cell lines HaCaT and normal human epidermal keratinocytes (NHEK) cells also resulted in decreased CPD removal upon UVB irradiation (Yang Z. et al., 2021). Interestingly, another study found that m^6^A was recruited to DNA damage lesions only in the presence of CPDs in response to UVA or UVB radiation, suggesting that m^6^A and m^6^A-associated enzymes may specifically recognize CPDs (Svobodová Kovaříková et al., 2020).

Furthermore, in response to UVA exposure, m^6^A RNA modifications may serve in the nucleotide excision repair pathway, but not the non-homologous end-joining (NHEJ) pathway, as treatment with an inhibitor of SUV40H1/H2, which are NHEJ-specific enzymes, had no effect on m^6^A recruitment (Svobodová Kovaříková et al., 2020). Interestingly, however, knockout of other NHEJ enzymes SUV39H1/H2, did decrease the recruitment of m^6^A in response to UVA exposure, suggesting that the role of m^6^A in NHEJ may be complex (Svobodová Kovaříková et al., 2020). In response to UVB exposure, METTL14 inhibited UVB-induced skin tumorigenesis by regulating global genome repair (GGR) in human keratinocyte cell lines (Yang Z. et al., 2021). Mechanistically, METTL14 regulated the m^6^A deposition onto the DNA damage repair gene *Ddb2* and promoted YTHDF1-mediated DDB2 translation, and subsequent knockdown of *Mettl14* resulted in decreased DDB2 abundance in an m^6^A-dependent manner (Yang Z. et al., 2021). The discrete epitranscriptomic mechanisms that underlie the DDR in response to UVA, UVB, and UVC exposure remains unclear.

Interestingly, in response to double-stranded breaks (DSBs), which were induced by X-ray radiation or Zeocin treatment, a chemical that induces DSBs, METTL3 was activated and phosphorylated at S43 by the key DDR kinase ATM, which then localized to DNA damage sites (Zhang C. et al., 2020). At these DNA-damage sites, METTL3 deposited m^6^A onto DNA damage-associated RNA, forming a DNA-RNA hybrid (Zhang C. et al., 2020). Accordingly, knockdown of *Mettl3* resulted in decreased homologous recombination, a key process in the double-stranded break repair process (Zhang C. et al., 2020). YTHDC1 was also recruited to the DNA-RNA hybrid sites, potentially serving to preserve m^6^A deposition on these RNA hybrids (Zhang C. et al., 2020). The DNA-RNA hybrids then recruited the DNA damage-associated proteins RAD51 and BRCA in order to initiate HR (Zhang C. et al., 2020).

Furthermore, in the NCCIT stem cell line, VIRMA was found to promote resistance to cisplatin through regulation of the DNA damage response (Miranda-Gonçalves et al., 2021). Accordingly, knockdown of VIRMA resulted in increased expression of DNA repair proteins, including γ H2AX, GADD45A ,and GADD45B, and promoted sensitivity to cisplatin (Miranda-Gonçalves et al., 2021). Whether VIRMA mediates the DDR in an m^6^A-dependent manner was not explored (Miranda-Gonçalves et al., 2021).

In addition to m^6^A writers, METTL16, a methyltransferase that targets non-coding RNAs, including U6 small nuclear RNA, was also recruited to sites of DNA damage at a later time point (20–30 mins post UVA/UVC micro-irradiation) (Svobodová Kovaříková et al., 2020). However, the substrates of METTL16 in response to UV radiation were not explored in this study (Svobodová Kovaříková et al., 2020).

#### Erasers

In response to metabolic stress, UVC, and H_2_O_2_ treatment, the m^6^A eraser FTO increased the mRNA stability of DNA repair pathway genes, including *Hspa1a* (*Hsp70*), in osteoblasts (Zhang Q. et al., 2019). Increased mRNA stability and expression of *Hspa1a* served to protect osteoblasts from NF-κ β-mediated apoptosis (Zhang Q. et al., 2019). While the *Hspa1a* mRNA transcript does contain m^6^A sites, this study did not address whether FTO promotes *Hspa1a* mRNA stability in an m^6^A-dependent manner (Zhang Q. et al., 2019).

**FIGURE 4:**
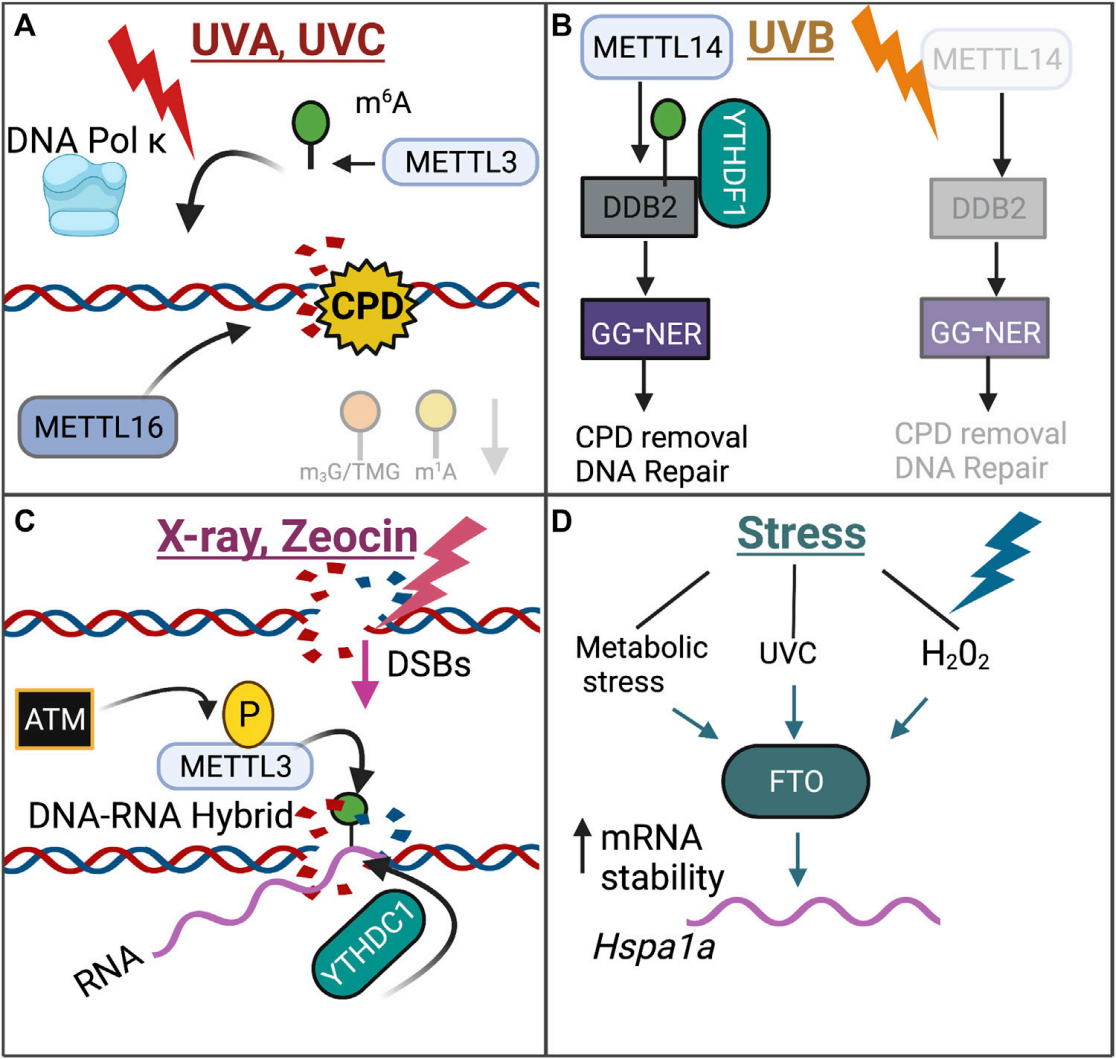
Review of the role of m^6^A within the DNA Damage Response. The role of m^6^A with the DNA Damage Response is dependent on the DNA damaging agent, highlighted through the distinct mechanisms that are employed upon exposure to UVA/UVC **(A)**, UVB **(B)**, X-ray/Zeocin **(C)**, or Stress **(D)**.

The role of m^6^A writers and erasers in the DNA damage response is highlighted in Figure 4.

The role of m^6^A in mediating DNA damage response is an emerging field of interest. There are several gaps in this field that should be addressed accordingly. One major area of interest is elucidating the specific roles of m^6^A readers in the DNA damage response. There is limited research on this topic. While m^6^A readers have been implicated in DDR, it has only been in the context of their communication with the m^6^A writers and erasers. Furthermore, another area of interest is to further elucidate the function of the m^6^A machinery in response to chronic vs. acute DNA damage in response to genotoxic agents such as UV radiation, arsenic, chemotherapy, and ionizing radiation.

### Liquid-Liquid Phase Separation

Liquid-liquid phase separation (LLPS) involves the formation of biological condensates containing aggregates of proteins or nucleic acids within the cell (Lyon et al., 2021). Biophysical mechanisms of liquid-liquid phase separation formation are discussed elsewhere (Lyon et al., 2021). Recent work has uncovered fascinating roles for these condensates in cellular functions, including involvement in stress responses, diseases, and aging (Alberti and Hyman, 2021; Lyon et al., 2021).

#### Readers

YTHDF readers YTHDF1 and YTHDF3 are believed to be critical in mediating LLPS as depletion of *Ythdf1* or *Ythdf3* prevented stress granule (SG) formation (Ries et al., 2019; Fu and Zhuang, 2020). There are contrasting reports on the role of YTHDF2 in LLPS and SG formation, as sodium-arsenite-induced SGs required YTHDF2, but not oxidative-stress-induced SG (Ries et al., 2019; Fu and Zhuang, 2020). These contrasting reports highlight that the role of YTHDF2 in LLPS and SG formation may be context-dependent (Ries et al., 2019; Fu and Zhuang, 2020). Biophysically, YTHDF1/3 are hypothesized to facilitate LLPS by lowering the activation energy input needed for phase separation (Fu and Zhuang, 2020). Alternatively, another hypothesis states YTHDF1/3 may serve as shell proteins that promote SG formation (Fu and Zhuang, 2020). However, the mechanism by which YTHDF1 and YTHDF3 mediate LLPs and SG formation is unclear (Fu and Zhuang, 2020). Furthermore, YTHDF3 has also been found to promote triaging of mRNAs into SGs in response to oxidative stress (Anders et al., 2018). Under these conditions, mRNA transcripts are dynamically patterned with m^6^A at the 5′-UTR and 5′-CDS regions and are partitioned into stress granules by YTHDF3, and are prevented from undergoing translation (Anders et al., 2018).

In addition to YTHDF1 and YTHDF3, two independent studies have established a role for YTHDC1 in LLPS. YTHDC1 is structurally made up of N or C-terminal internally-disordered regions (IDRs), which are believed to be necessary for YTHDC1’s role in LLPS (Lee J.-H. et al., 2021; Cheng et al., 2021). In MEF, 293T, and HeLa cells, m^6^A-eRNAs, which localize to active enhancer regions, recruited YTHDC1 to form YTHDC1-BRD4 condensates (Lee J.-H. et al., 2021). Additionally, YTHDC1 also formed m^6^A-YTHDC1 condensates, termed nYACs, in AML cells (Cheng et al., 2021). In this context, the number of nYACs increased in AML cells, as compared to normal hematopoietic cells, and also promoted tumorigenesis by promoting an undifferentiated state and cell survival (Cheng et al., 2021). Furthermore, nYACs can influence mRNA metabolism by preventing m^6^A-decorated mRNAs from being degraded by the PAXT-exosome complex (Cheng et al., 2021). The role of LLPS in tumorigenesis remains an emerging area of interest and is detailed further (Jiang et al., 2020).

It is important to note that whether m^6^A is critical for LLPS and stress granule formation remains controversial. One study found that m^6^A on mRNAs promoted YTHDF1-3 partitioning into phase-separated structures (Ries et al., 2019). However, a more recent study demonstrated that mRNAs, with or without m^6^A modifications, show minor differences in partitioning to stress granules and therefore argue that m^6^A may only play a minor role in stress granule partitioning (Khong et al., 2021). The authors of this study hypothesize that it is not only m^6^A-YTHDF interactions that promote stress granule partitioning, but rather, there may be several other RNA-protein interactions outside of m^6^A-YTHDF that promote stress granule portioning (Khong et al., 2021). The identity and nature of these RNA-protein interactions remain unclear.

The field of LLPS is rapidly expanding and research into this topic breaches disciplines in biophysics, biochemistry, disease biology, as well as epitranscriptomics. Of the many gaps of knowledge within this field, expanding the role of m^6^A machinery in LLPS, namely specific m^6^A writers and erasers, remains paramount.

**FIGURE 5:**
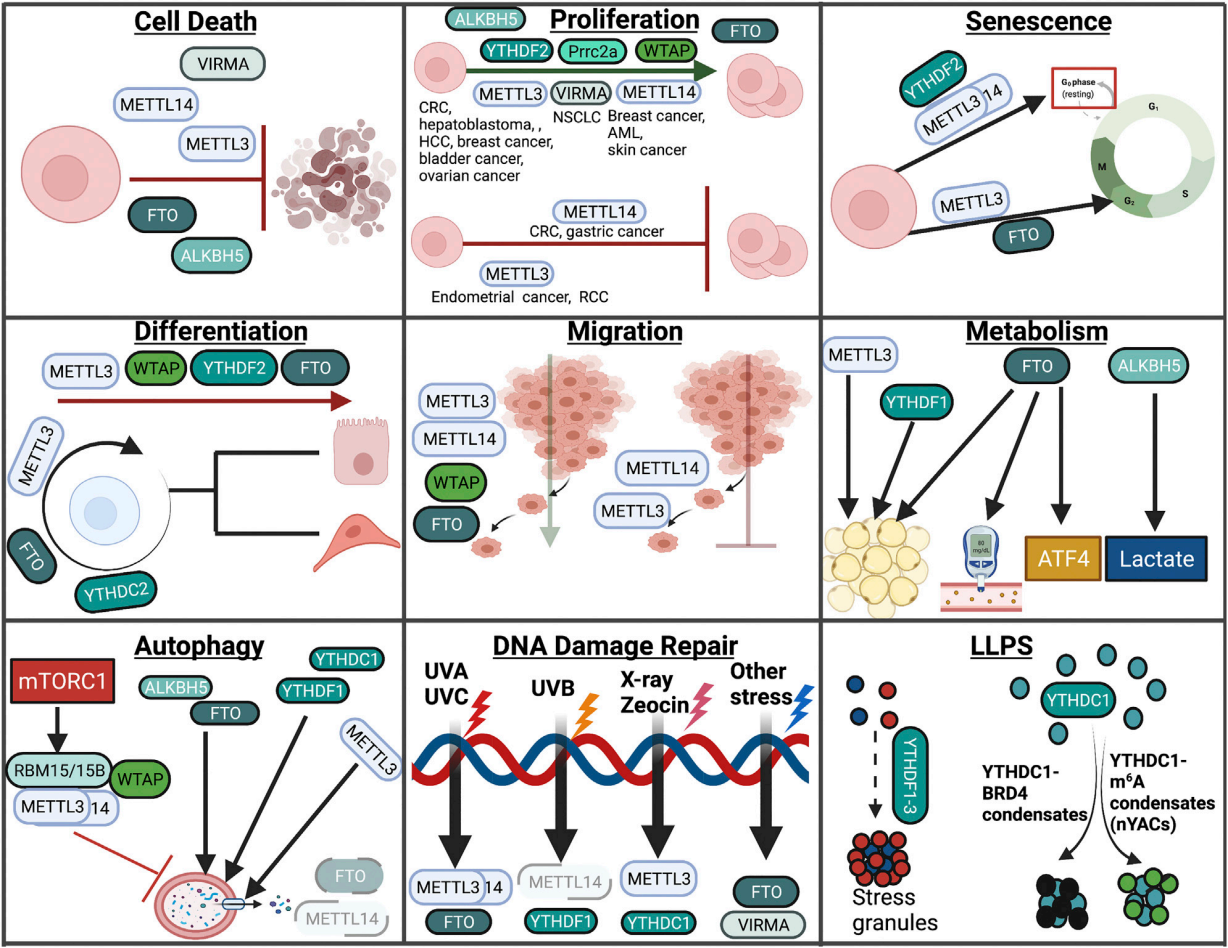
Overview of m^6^A in cellular processes. In this review, we highlight the role of RNA modifications in cellular processes such as cell death, proliferation, senescence, differentiation, migration, metabolism, autophagy, the DNA damage response, and LLPS. Within these processes, RNA modifications assume unique and context-dependent functions.

A summary of the role of m^6^A modification in cellular responses covered in this review can be found in Figure 5.

## The Role of m^5^C in Diverse Cellular Functions

**FIGURE 6:**
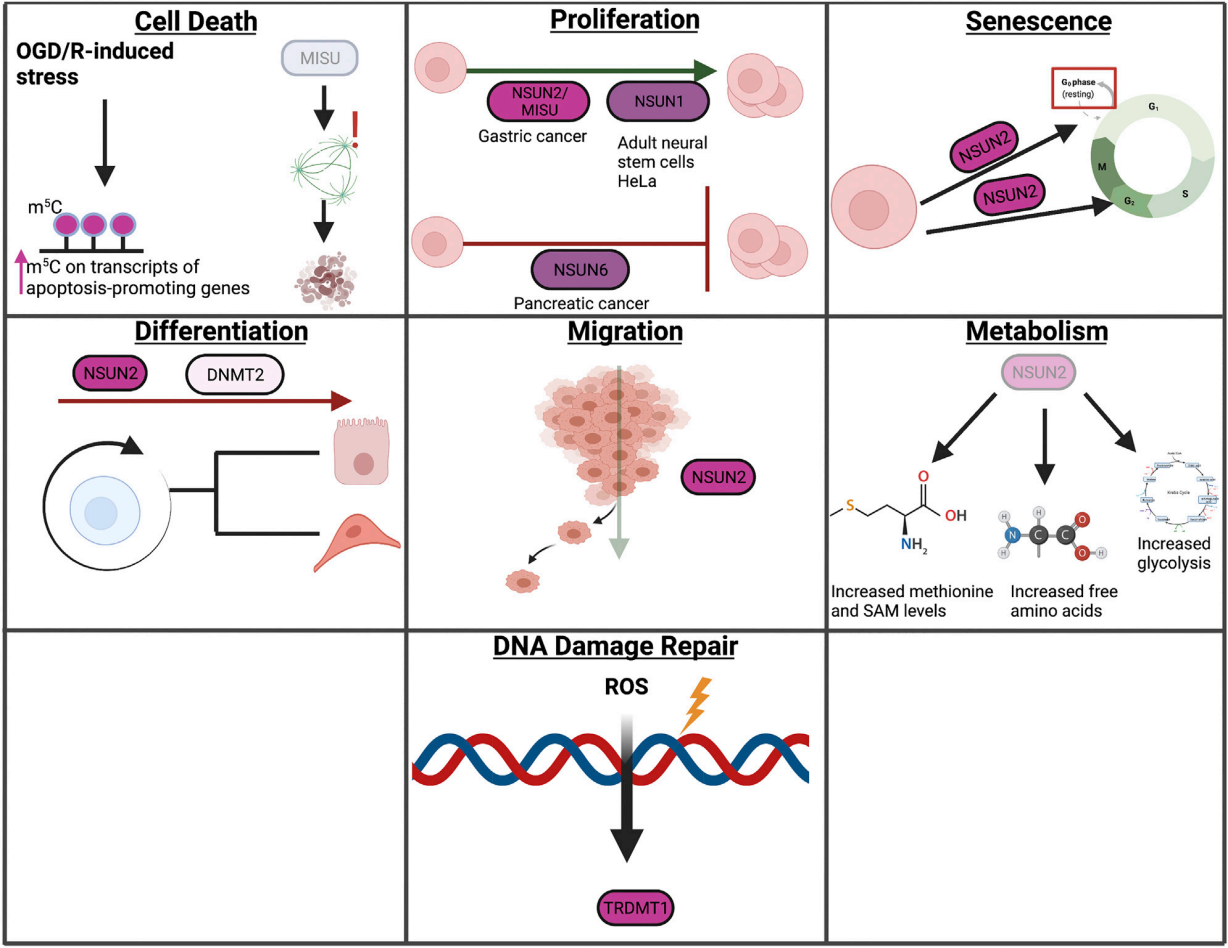
Overview of m^5^C in cellular processes. In this review, we highlight the role of RNA modifications in cellular processes such as cell death, proliferation, senescence, differentiation, migration, metabolism, and the DNA damage response. Within these processes, RNA modifications assume unique and context-dependent functions.

m^5^C has been implicated in several cellular contexts, including cell death, proliferation, senescence, differentiation, migration, metabolism, and DDR (Figure 6). The role of m^5^C in autophagy and LLPS has not been studied extensively and will therefore not be covered in this section. The role of m^5^C in autophagy and LLPS represents a gap of knowledge within this growing field and therefore requires further studies.

### Cell Death

#### Writers

The role of m^5^C in cell death is not widely explored. Accordingly, one study employing an oxygen-glucose deprivation/reoxygenation (OGD/R) model in neurons found that m^5^C-methylated sites were increased upon OGD/R (Jian et al., 2021). Furthermore, RNA bisulfite sequencing revealed that m^5^C hypermethylated transcripts after OGD/R were functionally involved in apoptosis (Jian et al., 2021). While the functional significance of these findings remains to be explored, these results suggest that m^5^C methylation may be important in mediating apoptosis in response to cellular stress mediated by OGD/R (Jian et al., 2021). Furthermore, MISU, an NSUN2 homolog, was identified as a critical regulator of mitotic integrity; accordingly, MISU depletion resulted in apoptosis, potentially through decreased spindle integrity and induction of aneuploidy (Hussain et al., 2009).

### Proliferation

#### Writers

m^5^C RNA methylation may also have important roles in cell proliferation. Similar to trends seen with m^6^A, the role of m^5^C RNA modifications in cell proliferation have been studied primarily in the context of cancer.

Low expression of NSUN6, an m^5^C methyltransferase, was found in pancreatic cancer patients, and may contribute to pancreatic cancer cell proliferation through regulation of CDK10 (Yang R. et al., 2021). While decreased NSUN6 expression was correlated with decreased CDK10 expression, resulting in increased cell proliferation, this study did not establish whether NSUN6 regulates CDK10 expression in an m^5^C-dependent manner (Yang R. et al., 2021).

High levels of NSUN2, another m^5^C methyltransferase, have been found in several different cancer types, but the functional role of NSUN2 in regulating proliferation was not clear (Okamoto et al., 2012). A recent study found in gastric cancer found that high NSUN2 levels were associated with worse overall survival, and that knockdown of *Nsun2* resulted in decreased proliferation in gastric cancer cells (Hu et al., 2021). NSUN2 protein stability was found to be regulated by SUMOylation, a post-translational modification (Hu et al., 2021). Interestingly, increased proliferation was also noted upon *Nsun2* overexpression in these cells, using both wild-type and catalytically inactive mutant NSUN2, suggesting that NSUN2 may have both m^5^C-dependent and m^5^C-independent functions in promoting proliferation (Hu et al., 2021). RNA bisulfite sequencing revealed that NSUN2-dependent m^5^C methylated transcripts were involved in oncogenic pathways, including the RAP1 pathway, as well as pathways involved in drug resistance and cell cycle (Hu et al., 2021). Furthermore, NSUN2 promoted proliferation in U2OS cells by depositing m^5^C onto *Cdk1* transcripts, resulting in increased CDK1 translation (Xing et al., 2015). The role of NSUN2 in promoting proliferation is further detailed elsewhere (Wang, 2016).

Interestingly, MISU, a NSUN2 homolog, was identified as a MYC target and mediated MYC-induced cell growth in human epidermis cells (Frye and Watt, 2006). However, the mechanism by which MISU regulates MYC-induced proliferation is unclear (Frye and Watt, 2006).

Furthermore, the expression of NSUN1, alternatively known as NOP2, was increased in adult neural stem cells after stroke and was positively correlated with adult neural stem cell proliferation, suggesting a potential role for NSUN1 in promoting recovery after stroke (Kosi et al., 2015). Additionally, NSUN1, also known as NOL1, promoted proliferation by binding to the *Ccnd1* promoter and promoting *Ccnd1* transcription in HeLa cells (Hong et al., 2016). Whether NSUN1 promoted the proliferation in an m^5^C-dependent manner in these studies was not established.

### Senescence

#### Writers

The m^5^C writer NSUN2 has been found to promote senescence in a variety of contexts. In HeLa cells, METTL3/14 and NSUN2 cooperated to increase p21 translation in response to oxidative stress, ultimately leading to the induction of cellular senescence (Li Q. et al., 2017). NSUN2 also promoted oxidative-stress-induced cellular senescence in human umbilical vein endothelial cells through m^5^C methylation of *Shc* mRNA, which led to increased SHC protein expression, activation of p38/MAPK, and increased ROS levels, thereby establishing a positive feedback loop (Cai et al., 2016). However, the role of NSUN2 in regulating senescence may be context and stimuli-dependent. In human diploid fibroblasts, NSUN2 negatively regulated senescence by methylating *p27* ^*KIP1*^, a CDK inhibitor, at the 5′-UTR, resulting in decreased p27 translation and increased CDK1 translation (Tang et al., 2015). By indirectly promoting CDK1 translation, NSUN2 served to promote cellular proliferation and inhibit cellular senescence (Tang et al., 2015).

### Differentiation

#### Writers

NSUN2 levels are highly expressed in undifferentiated epidermal progenitor cells (Sajini et al., 2019). Vault tRNAs (vtRNAs) are RNA POLIII-derived transcripts that make up vault ribonucleoproteins, and can be processed into smaller regulatory RNAs (svRNAs) (Stadler et al., 2009; Sajini et al., 2019). Accordingly, *Sajini* et al. found that processing of vtRNA VTRNA1.1 is dependent on NSUN2-dependent m^5^C methylation and is critical for proper epidermal cell development (Sajini et al., 2019). NSUN2-mediated m^5^C on tRNAs is also believed to be required for epidermal stem cell, testis, and neural stem cell differentiation (Blanco et al., 2011; Hussain et al., 2013; Flores et al., 2017). Due to the important role of NSUN2 in promoting neural stem cell differentiation, loss of *Nsun2* is linked to several developmental disorders (Flores et al., 2017). DNMT2-mediated m^5^C on tRNA is believed to be required for hematopoiesis, as *Dnmt2*-deficient mice showed decreased stem and progenitor cell populations (Tuorto et al., 2015). The role of m^5^C in differentiation and development is summarized elsewhere (Song et al., 2021).

### Migration

#### Writers

In addition to m^6^A writers, the m^5^C writer NSUN2 promoted the mRNA translation of autotaxin (*Atx*) in U87 glioma cells in an m^5^C-dependent manner (Xu et al., 2020). NSUN2 deposited m^5^C at the 3′-UTR of *Atx*, enhancing *Atx* translation and promoting the export of *Atx* from the nucleus to the cytoplasm through coordination with m^5^C reader ALYREF (Xu et al., 2020). Downstream, ATX then converts lysophosphatidylcholine to lysophosphatidic acid, a lipid that has been to promote migration and overall tumorigenicity (Valdés-Rives and González-Arenas, 2017; Xu et al., 2020). Furthermore, NSUN2 was also found to promote migration in gastric cancer cells (Hu et al., 2021).

### Metabolism

#### Writers

*Nsun2* ^*−/−*^ mice resulted in changes in the metabolism of methionine and amino acids, and the TCA cycle (Gkatza et al., 2019). *Nsun2* deletion resulted in increased methionine and S-adenosyl-methionine (SAM) levels (Gkatza et al., 2019). Furthermore, free amino acid levels were increased upon loss of *Nsun2*, which was hypothesized to indicate overall decreases in translation (Gkatza et al., 2019). Additionally, *Nsun2* loss resulted in a metabolic shift towards glycolysis (Gkatza et al., 2019). Taken together, the authors hypothesized that *Nsun2* loss results in the induction of a catabolic state, and that NSUN2 functions to promote an anabolic fate (Gkatza et al., 2019).

### DNA Damage Response

#### Writers

Interestingly, *Chen* et al. found that m^5^C was localized to DSBs upon ROS-induced DNA damage, and is present at DNA-damage-induced DNA-RNA hybrids (Chen H. et al., 2020). Interestingly, tRNA methyltransferase TRDMT1 was also found to localize to DNA-damage-induced DNA-RNA hybrids and was hypothesized to serve as a damage-induced m^5^C methyltransferase (Chen H. et al., 2020). Together, TRDMT1 and m^5^C are believed to be necessary to mediate homologous recombination in response to DNA damage (Chen H. et al., 2020; Zhu X. et al., 2021).

## The Role of m^1^A in Diverse Cellular Functions

**FIGURE 7:**
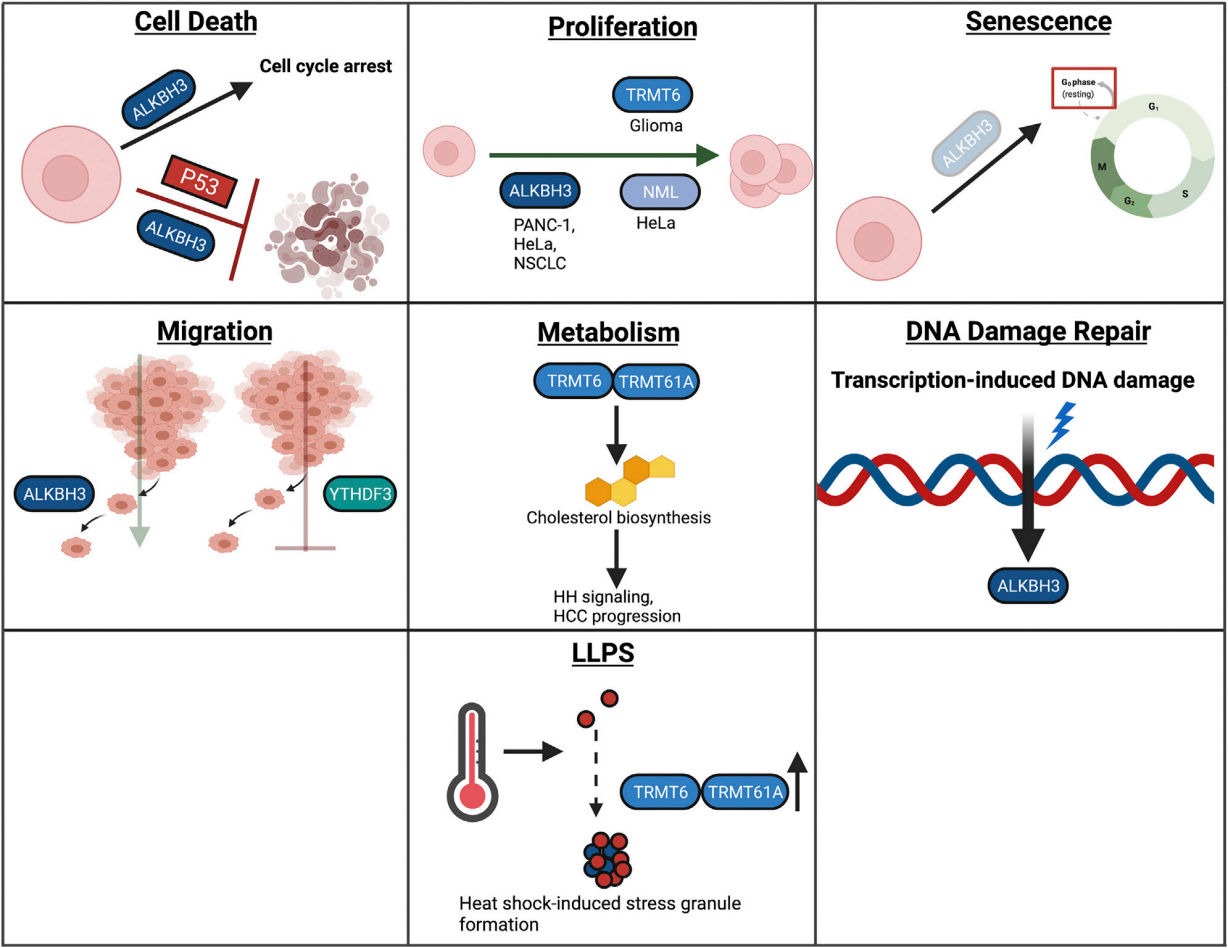
Overview of m^1^A in cellular processes. In this review, we highlight the role of RNA modifications in cellular processes such as cell death, proliferation, senescence, migration, metabolism, the DNA damage response, and LLPS. Within these processes, RNA modifications assume unique and context-dependent functions.

m^1^A has been studied in several cellular contexts, including, cell death, proliferation, senescence, migration, metabolism, DDR, and LLPS (Figure 7). The role of m^1^A in differentiation in autophagy has not been studied extensively and will therefore not be covered in this section. The role of m^1^A in differentiation and autophagy requires further study.

### Cell Death

#### Erasers

In NSCLC, knockdown of the m^1^A eraser *Alkbh3* promoted cell cycle arrest (Kogaki et al., 2017). Interestingly, knockdown of both *Alkbh3* and *Tp53* resulted in the induction of apoptosis, suggesting that TP53 may be critical for shifting cell fate from cell cycle arrest to undergoing apoptosis (Kogaki et al., 2017). However, whether ALKBH3 mediates this function as an RNA or DNA methyltransferase was not elucidated (Kogaki et al., 2017).

### Proliferation

#### Writers

Many studies have found positive associations with m^1^A regulators, such as the m^1^A methyltransferase TRMT6, and cancer (Wang et al., 2019; Shi et al., 2020; Wang B. et al., 2021). However, few studies have established the biological mechanisms by which m^1^A regulators assert their oncogenic function. Inhibition of *Trmt6* resulted in decreased proliferation in glioma cell lines, establishing a potential oncogenic role for TRMT6 in regulating proliferation (Macari et al., 2016; Wang B. et al., 2021). Furthermore, inhibition of *Alkbh3*, an m^1^A demethylase, resulted in decreased proliferation in HeLa, PANC-1, and NSCLC cancer cells, suggesting a potential role for this demethylase in proliferation (Tasaki et al., 2011; Ueda et al., 2017; Chen et al., 2019). Furthermore, *Waku* et al. established that nucleomethylin (NML) can function as an m^1^A 28S rRNA methyltransferase, and that inhibition of NML results in decreased proliferation (Waku et al., 2016).

### Senescence

#### Erasers

Few studies have explored the role of m^1^A in cellular senescence. One study, however, found that knockdown of *Alkbh3* in NSCLC cells resulted in senescence induction and cell cycle arrest, followed by increased expression of cell cycle arrest proteins, p27 and p21 (Tasaki et al., 2011).

### Migration

#### Erasers

Few studies have investigated the role of m^1^A and m^1^A regulators in migration. One study found that knockdown of *Alkbh3* in HeLa cells resulted in decreased invasion (Chen et al., 2019). Furthermore, YTHDF3, serving as an m^1^A reader, was found to inhibit invasion in HTR8/SVneo cells by promoting the mRNA decay of *Igf1r* (Zheng et al., 2020). IGF1R is upstream of the pro-migratory MMP9 signaling pathway, and subsequent knockdown of *Ythdf3* resulted in increased IGF1R and MMP9 expression, resulting in increased invasion and migration in these cells (Zheng et al., 2020).

### Metabolism

#### Writers

As previously mentioned, TRMT6 has been found to be associated with oncogenesis in a variety of different cancers. In HCC, the TRMT6/TRMT61A m^1^A methyltransferase complex was identified to mediate m^1^A tRNA methylation, which resulted in increased PPAR δ translation and cholesterol biosynthesis (Wang Y. et al., 2021). Increased cholesterol biosynthesis, in turn, activated Hedgehog signaling and promoted the formation of liver cancer stem cells and HCC tumorigenesis (Wang Y. et al., 2021).

### DNA Damage Response

#### Erasers

Knockdown of *Alkbh3* in NSCLC resulted in increased phosphorylation of critical DDR factors ATM, ATR, and H2AX, suggesting that decreased ALKBH3 may promote single or double-stranded breaks (Kogaki et al., 2017). These DDR factors, as well as DNA-PKcs, were further upregulated in *p53/Alkbh3* dual-knockout cells, establishing that p53 may be a critical regulator of ALKBH3 in mediating DDR (Kogaki et al., 2017). Whether the role of ALKBH3 in DDR is mediated through its demethylase function was not explored in this study (Kogaki et al., 2017). ALKBH3 has also been suggested to function as a DNA repair protein in response to transcription-induced DNA damage (Liefke et al., 2015). Furthermore, levels of m^1^A, found on small RNAs, were also noted to be decreased in UV-irradiated cells (Svobodová Kovaříková et al., 2020). However, the functional role of m^1^A on small RNAs in response to UV exposure was not detailed in this study (Svobodová Kovaříková et al., 2020).

### LLPS

#### Writers

m^1^A methyltransferase TRMT61/61A and m^1^A were increased in heat-shock-induced, stress-granule-sequestered mRNAs (Alriquet et al., 2020). In response to stress, mRNAs can form irreversible protein aggregates (Alriquet et al., 2020). Conversely, m^1^A-patterned mRNAs were identified to be sequestered into reversible mRNA-protein aggregates, which can then undergo translation (Alriquet et al., 2020). Therefore, the authors hypothesize that m^1^A serves a protective role on mRNAs in response to stress (Alriquet et al., 2020).

## Therapeutics Targeting RNA Modifications

Due to the pervasiveness of RNA modifications in disease, the development of targeted therapeutics remains critical and is an active area of research. Here, we will briefly summarize advances in the development of therapeutics targeting RNA modifications.

### m^6^A-Targeted Therapeutics

*Yankova* et al. recently identified a small molecule inhibitor (STM2457) for METTL3 using a high throughput drug screen (Yankova et al., 2021). STM2547 was identified to be specific to METTL3 and did not disrupt the METTL3-METTL14 complex (Yankova et al., 2021). As METTL3 has been found to serve an oncogenic function in leukemia, the *in vitro* and *in vivo* efficacy of STM457 was explored as a therapeutic for AML (Vu et al., 2017). STM2457-treatment in AML cell lines resulted in decreased proliferation in a dose-dependent manner and decreased the colony forming capability of mouse AML cells (Yankova et al., 2021). Interestingly, STM2457 showed selectivity for AML cells, but did not affect CD34^+^ cells, hematopoietic stem and progenitor cells, or non-leukemogenic cell lines (Yankova et al., 2021). STM2457 also decreased the protein expression of oncogenic METTL3 targets, SP1 and BRD4 (Yankova et al., 2021).

Many small molecule inhibitors for FTO have been discovered, including rhein, NCDPCB, meclofenamic acid, MO-I-500, and fluorescein derivatives, among others (Chen et al., 2012; Wang T. et al., 2015; He et al., 2015; Huang et al., 2015; Singh et al., 2016). While these inhibitors inhibit FTO, clinical efficacy of FTO inhibitors has remained unclear. FTO has been found to serve as an oncogene in AML (Li Z. et al., 2017). Accordingly, two studies have developed FTO inhibitors targeting AML (Huang et al., 2019; Su et al., 2020). *Huang* et al. identified FB23-2 as a potential inhibitor for FTO (Huang et al., 2019). Treatment of AML cell lines for FB23-2 slightly decreased AML proliferation and promoted apoptosis, as well as promoted myeloid differentiation (Huang et al., 2019). FB23-2 treatment also resulted in minimal changes in proliferation in bone marrow cells derived from a healthy donor (Huang et al., 2019). Furthermore, FB23-2 showed promising therapeutic efficacy in mice, targeting both AML and leukemia stem cell populations (Huang et al., 2019). Furthermore, using a high throughput screen, *Su* et al. reported the discovery of two small molecule inhibitors targeting FTO, CS1 and CS2, with efficacy in targeting AML (Su et al., 2020). Treatment of AML cell lines with CS1 and CS2 resulted in decreased proliferation, increased apoptosis, and prevented the self-renewal capabilities of leukemia stem cells and leukemia initiating cells (Su et al., 2020). Treatment of healthy control cells showed no change (Su et al., 2020). To date, neither FB23-2 or CS1/CS2 have been employed in clinical trials. While *Selberg* et al. have described the development of a potential ALKBH5 inhibitor, further studies are needed to reconcile the cell-type specific effect of ALKBH5 inhibition (Selberg et al., 2021).

### m^5^C-Targeted Therapeutics

m^5^C-directed therapeutics have also been explored. Few studies have explored the therapeutic potential of targeting m^5^C reader YBX1, but have only identified non-specific compounds that effectively target YBX1 (Shibata et al., 2020). *Shibata* et al. identified compounds, TAS0612 and everolimus, as potential compounds that target YBX1 phosphorylation (pYBX1) (Shibata et al., 2020). Increased YBX1 phosphorylation was found to be associated with resistance to fulvestrant, an antiestrogen commonly used to treat ER-positive breast cancer (Shibata et al., 2020). TAS0612 is a multi-kinase inhibitor that targets both the AKT/mTOR/p70S6K pathway, and pYBX1 was identified to be a downstream target of these pathways (Shibata et al., 2020). Everolimus is an mTORC1 inhibitor (Shibata et al., 2020). Accordingly, TAS0612 and everolimus treatment resulted in increased sensitivity to fulvestrant (Shibata et al., 2020). However, this study did not address whether changes in YBX1 phosphorylation changes m^5^C regulation (Shibata et al., 2020). Azacytidine is a well-established drug targeting DNMT2 DNA methylation (Stresemann and Lyko, 2008). However, one study, using bisulfite sequencing, argued that azacytidine may also target DNMT2-mediated tRNA methylation (Schaefer et al., 2009). However, follow-up studies are needed to identify whether azacytidine-mediated changes in tRNA methylation are due to m^5^C or other mechanisms (Schaefer et al., 2009).

### m^1^A-Targeted Therapeutics

*Wang* et al. recently identified thiram as a potential candidate compound that selectively inhibits m^1^A writer complex TRMT6/TRMT61A (Wang Y. et al., 2021). Thiram treatment resulted in decreased oncosphere formation in HCC cell lines *in vitro*, and decreased tumor growth *in vivo* (Wang Y. et al., 2021). However, further pre-clinical studies are necessary to determine the safety of thiram treatment in patients, due to reported toxicities (Maita et al., 1991; Wang Y. et al., 2021).

Compound HUHS015 has been identified as an ALKBH3 inhibitor (Nakao et al., 2014). As previously mentioned, ALKBH3 has been found to serve an oncogenic role in many cancers, including prostate cancer (Liefke et al., 2015). HUHS015 has been found to decrease the growth of prostate cancer cell line DU145 and decreased tumor burden in xenograft models (Nakao et al., 2014; Mabuchi et al., 2015). To date, no clinical trial using HUHS015 has been employed.

## Perspectives

While the roles of RNA modifications have been extensively studied for several cellular functions, there remain several areas of interest that are not well-established and require further examination.

Two areas of interest that remain open areas of research include evaluating the roles of RNA modifications in mediating specialized forms of cell death and within LLPS. In the area of cellular death, the role of RNA modifications has been well-studied in terms of apoptosis. However, emerging evidence suggests that RNA modifications may be important in mediating specialized forms of cell death including ferroptosis, pyroptosis, or other mechanisms of specialized cell death (Guo et al., 2020; Shen et al., 2021). Understanding the roles of RNA modifications in these specialized forms of cell death may lead to increased knowledge surrounding the cellular decisions that mediate these forms of cell death.

Furthermore, as previously mentioned, LLPS remains an emerging field of research. The field of LLPS encompasses the intersection of cell biology and biophysics; not only are the biophysical mechanisms by which these condensates form an active area of interest, but more recently, increased attention has been placed on detailing the role these condensates play within cellular processes. Increasing our understanding of RNA modifications in this process will aid in understanding the function and necessity of LLPS in mediating cellular functions.

The roles of other RNA modifications, other than m^6^A, within mammalian cellular processes is another gap of knowledge within the field of epitranscriptomics that remains critical to address. For example, while there are several studies that have identified cellular functions for pseudouridine in *Drosophila*, few studies have been done to explore the role of pseuoduridine in mammalian cellular functions (Vicidomini et al., 2015; Song et al., 2020).

Another area of interest that requires further study is understanding the cell-type specific function of the role of RNA modification in cellular functions. As demonstrated, not only do RNA modifications differ across cell types, but they can also differ across contexts, including across differentiation states and in response to stress. Understanding the relevance and pervasiveness of RNA modifications in these processes, and how different cell types adopt distinct mechanisms for RNA modifications across these functions, remains an important area of research.

In addition, due to the prevalence of RNA modifications in diverse cellular functions, the dysregulation of RNA modifications contributes to the etiology of several diseases. RNA modifications have been found to contribute to the pathologies of several diseases including cancer, diabetes, cardiovascular diseases, and developmental and neurological diseases. Increasing our understanding of the distinct roles that RNA modifications play in these cellular processes will allow for an increased understanding of disease etiology. While there are no therapeutics currently in clinical use that target RNA modifications, an increased understanding of their roles in disease etiology may contribute to the development of therapeutics that aim to selectively target this epitranscriptomic re-wiring.
